# Storage Stability Enhancement of Lactic Acid Beverage Using Anti-MDA *Lactiplantibacillus plantarum* NJAU-01: The Antioxidant’s Role

**DOI:** 10.3390/foods14010052

**Published:** 2024-12-27

**Authors:** Yuehao Wu, Menghao Zhao, Suyun Li, Siyu Liu, Song Gao, Rui Liu, Mangang Wu, Hai Yu, Qingfeng Ge

**Affiliations:** College of Food Science and Engineering, Yangzhou University, Yangzhou 225127, Chinags@yzu.edu.cn (S.G.); ruiliu@yzu.edu.cn (R.L.); mgwu@yzu.edu.cn (M.W.); yuhai@yzu.edu.cn (H.Y.)

**Keywords:** malondialdehyde, *Lactiplantibacillus plantarum* NJAU-01, beverages, antioxidant, storage

## Abstract

This study evaluated the inhibitory efficacy of *Lactiplantibacillus plantarum* NJAU-01 (NJAU-01) on oxidation associated with malondialdehyde (MDA) and utilized the bacteria in a functional lactic acid beverage. The antioxidant capacity of the bacteria was measured in vitro, the production conditions (inoculum, fermentation time, and sugar addition) of the lactic acid beverage were optimized, and the effects of NJAU-01 on antioxidant, flavor profile, and storage stability of lactic acid beverages were investigated. The results revealed that NJAU-01 exhibited a high tolerance towards MDA at 40 mM, and that it also exhibited outstanding antioxidant capacity in vitro and antioxidant enzyme activity throughout its growth stage. The beverage demonstrated an elevated antioxidant capacity and efficiently eliminated MDA. Additionally, the NJAU-01 lactic acid beverage could be stored at 4 °C for 21 days, exhibiting stable sensory attributes and strong resistance against lipid peroxidation. The study yielded insights into the role of NJAU-01 in improving the storage stability of lactic acid beverages thereby contributing to a deeper understanding of the specific mechanisms by which probiotics enhance beverage quality. These findings can facilitate a more effective utilization of this knowledge in the food industry.

## 1. Introduction

Public interests in probiotic-infused consumer products are steadily rising [1]. The potential of probiotics in disease prevention and treatment has attracted the attention of researchers worldwide, and the commercial application of probiotic-fermented foods is prevalent in the functional food industry. Many studies have indicated that probiotics are effective in alleviating various metabolic disorders and enhancing physical functions, particularly in managing associated ailments, such as obesity, type II diabetes, hypertension, and irritable bowel syndrome [2]. These numerous benefits have contributed to the growing popularity of probiotics. A comprehensive investigation into the functionalities of various strains of probiotics will establish a theoretical basis for the advancement of the functional food industry.

The microorganism *Lactobacillus*, widely recognized as a safe, probiotic organism, has numerous applications in the food industry [3]. Extensive research has discovered that certain strains of *lactobacillus* not only enhance the sensory and nutritional characteristics of products, but also improve food safety and quality. Notably, some strains exhibited remarkable antioxidative properties both in vivo and in vitro. Amanatidou A et al. [4] found that *Lactobacillus rhamnosus* GG exhibited significant abilities in iron chelation, superoxide anion scavenging, and the inhibition of lipid peroxidation. In addition, *Lactobacillus paracasei* Fn032, *Lactobacillus rhamnosus* GG, and *Lactobacillus* spp. Fn 001 in a simulated colonic microbiota system indicated the ability to attenuate hydroxyl radical production [5]. Some studies have indicated that the oral administration of live recombinant *lactobacillus* strains producing superoxide dismutase (SOD) could ameliorate TNBS-induced colitis in mice, and enhance the activities of catalase (CAT), SOD, and glutathione peroxidase (GSH-Px) in the serum, liver, and intestinal tract of mice, as well as reduce the levels of MDA [6]. Although the antioxidant activities of numerous Lactobacillus were demonstrated, the underlying mechanisms behind these activities remain largely unknown.

Lactic acid beverages, as a type of easily digestible probiotic product that is rich in nutrients, were extensively studied and widely applied in global markets. In recent years, diverse models like the in vitro elimination of reactive oxygen species, cell experiments, and animal experiments were developed to investigate the antioxidant effects of lactic acid bacteria. Based on this, Wang et al. [3] summed up the eight major ways through which probiotics exert antioxidant effects. The companies producing these beverages have developed unique brands based on distinctive fermentation strains [7]. The diverse strains contribute to the unique functionalities and flavor profiles of lactic acid beverages. The fermentation of lactic acid beverages using lactic acid bacteria was employed in certain studies to exert antibacterial [8] and antioxidant [9] effects. Various protein oxidation and lipid oxidation events can compromise the functional properties and flavor quality of lactic acid beverages during storage at 4 °C. Malondialdehyde (MDA), a secondary product of polyunsaturated fatty acid peroxidation, is widely acknowledged as a significant biomarker of oxidative stress [10]. MDA not only directly induces oxidative stress but also generates a considerable amount of reactive oxygen species (ROS). These ROS can attack biological macromolecules such as DNA, proteins, and lipids, leading to nucleic acid carboxylation, protein denaturation, and lipid peroxidation, ultimately resulting in cellular dysfunction [11]. Consequently, it is imperative to identify effective strategies to mitigate oxidative stress induced by MDA.

A previous study discovered a decline in viability of *Lactobacillus acidophilus* in yogurt during storage, which was accompanied by a reduction in DPPH scavenging activity after 14 days [12]. Our previous study has proved that *Lactiplantibacillus plantarum* NJAU-01 indicated capabilities of acid production [13], antioxidation [14,15,16], and regulation of intestinal microbiota [17], but the storage stability of lactic acid beverages supplemented with NJAU-01 has not been extensively studied.

Lactic acid bacteria usually produces antioxidant compounds like glutathione (GSH), butyrate, and folic acid. The study by Kullisaar et al. [18] revealed *L. fermentum* E-3 and E-18 could generate a high amount of GSH. Kusuhara et al. [19] determinedthat *Streptococcus thermophilus* YIT 2001 mitigates oxidative stress in hosts through GSH as an active antioxidant component. Cataldo et al. [20] demonstrated that *Lactobacillus brevis* CRL 2013, which produces a high yield of γ-aminobutyric acid, could effectively alleviate the inflammatory responses in lipopolysaccharide-induced mice. The antioxidant activity of lactic acid bacteria might also be attributed to the antioxidant enzymes they possess. Li et al. [21] verified that some superior antioxidant probiotics, such as *Weissella hellenica* L23, could significantly enhance GSH-Px activity in the liver of aging model rats, as well as GSH-Px, T-SOD activity, and total antioxidant capacity (T-AOC) in the serum. Moreover, *Lactobacillus plantarum* L9 could significantly reduce MDA content in aging model rats [22]. However, the deep antioxidative mechanism of NJAU-01 in beverages is still unknown.

Currently, the lack of knowledge on anti-lipid oxidation properties of lactic acid beverages poses the urgent need for the elucidation of resistance to lipid oxidation, which is crucial for informing the industrial processing and storage techniques employed for lactic acid beverages.

This research investigated the alterations in indices of in vitro antioxidants and antioxidant enzymes of NJAU-01 under MDA stress. Inoculum, fermentation time, and sugar addition were identified as three critical factors. Sensory evaluation and total antioxidant capacity were utilized as response variables, and response surface methodology (RSM) was employed to optimize the formulation of the NJAU-01 lactic acid beverage, evaluating their capacity to eliminate MDA and the quality of storage.

## 2. Materials and Methods

### 2.1. Strain Preparation

*Lactiplantibacillus plantarum* NJAU-01 (CGMCC14194), screened from traditional dry-cured Jinhua ham [13], was stored (OD_600_ 1.6, 10^8^ CFU/mL) in MRS broth (Bio-way Technology Co., Ltd., Shanghai, China) that was mixed with glycerol (50%, *v*/*v*) at frozen (−80 °C) conditions. Previous experiments comparing NJAU-01 with the commercially available strains *Lactobacillus rhamnosus* LGG and *L. plantarum* JMZ-12, used as positive controls (Figure S1), demonstrated that NJAU-01 possesses a high degree of tolerance to MDA. The strains were activated twice and inoculated in 50 mL MRS broth (1%, *v*/*v*) at 37 °C for 18 h for further use.

### 2.2. Preparation and Content Determination of Malondialdehyde (MDA)

MDA was prepared following the protocol of Wu et al. [23], with minor adjustments. In total, 21 mL of 1,1,3,3-tetraethoxypropane (TEP), 25 mL of HCL (5 M), and 79 mL of deionized water were added to a beaker under dark conditions. The mixture was stirred in a water bath at 45 °C for 30 min, followed by cooling to room temperature. Subsequently, the pH was adjusted to 7.0 using NaOH (6 M). The concentration of MDA was determined by reacting it with thiobarbituric acid (TBA). Specifically, the absorption value of the 1 mL sample and 1 mL mixed solution (0.2 M TCA, 1.5 mM EDTA, 0.02 M TBA) was determined at 532 nm (Infinite M200 Pro Tecan laboratory equipment Co., Ltd. Shanghai, China) after 90 °C (water bath) for 30 min. The MDA content was quantified using a standard curve established through TEP analysis.

MRS broth supplemented with 40 mM MDA was inoculated with the twice-activated NJAU-01 strain (1%, *v*/*v*) and incubated at 37 °C for subsequent experimental evaluations. The consumption rate was calculated by dividing the time difference between the two malondialdehyde contents.

### 2.3. Kinetic Growth

The growth curve of NJAU-01 under 40 mM MDA stress was performed in accordance with Tang et al. [24], with slight modifications. The strains mentioned in Section 2.1 were inoculated to 300 mL MRS broth supplemented with 40 mM MDA. The OD_600_ was measured every 2 h by removing 200 μL bacterial mixture into a 96-well plate in a multimode microplate reader (Infinite M200 Pro Tecan laboratory equipment Co., Ltd., Shanghai, China), and the growth curve was plotted.

### 2.4. Preparation of Cell-Free Extracts of NJAU-01

The NJAU-01 bacterial suspension was previously observed to exhibit tolerance at a maximum concentration of 40 mM MDA, and its growth curve was obtained (Figure S1). Based on the growth curve, the specific time points were selected for conducting further analysis, in comparison to NJAU-01 under non-MDA stress: 0 h, 4 h (early lag phase), 12 h (middle lag phase), 24 h (end lag phase), 26 h (early log phase), 32 h (middle log phase), and 44 h (stable phase). The activated bacterial solution described in Section 2.1 was inoculated in 300 mL MRS broth with 40 mM MDA (1%, *v*/*v*) at 37 °C.

In our previous research, we examined the antioxidant capacity of cell-free extracts from NJAU-01 using various cell disruption techniques [25]. Cell-free extracts at each stage were prepared using a modified method of Li et al. [26], involving lysozyme combined with ultrasound treatment. Specifically, 9 mL of bacterial suspension (OD_600_ 1.6) was mixed with 1 mL of lysozyme (20,000 U/mg, Sangon Biotech Co., Ltd., Shanghai, China), with a final concentration of 3.24 mg/mL. The mixture was incubated at 37 °C for 60 min, followed by ultrasonic disruption in an ice bath for 50 min (ultrasound 20 s, intermittent 10 s, ultrasonic power 220 W, cycle 100 times). Afterwards, the samples were centrifugated at 6000× *g*, and the resulting supernatant was filtered through a 0.45 μm PES Syringe Filter (ALWSCI Technology Co., Ltd., Shaoxing, China) to obtain the cell-free extract for subsequent determinations.

### 2.5. Determination of Oxidation and Antioxidant Indices of Cell-Free Extracts

#### 2.5.1. Schiff Base

The Schiff base assay was performed referring to the method described by Padilla et al. [27], with slight modifications. A sample (protein content 1.2 mg/mL) was dissolved in 100 mM sterile PBS buffer (containing 8 M urea, pH = 7) at a ratio of 1:20. The excitation wavelength for fluorescence scanning on the microplate reader was set at 350 nm, and the emission wavelength was set at 330–380 nm. Both the excitation and emission slit widths were set at 10 nm (Infinite M200 Pro Tecan laboratory equipment Co., Ltd. Shanghai, China), and the results were expressed as fluorescence intensity.

#### 2.5.2. Determination of Total Sulfhydryl and Disulfide Bond Content

The determination of total sulfhydryl groups (TSH) and disulfides in cell-free extracts was conducted according to the method described by Ge et al. [17], with some modifications. In total, 1.0 mL of a cell-free extract was mixed with 0.9 mL of urea buffer (containing 10 mM EDTA, 0.6 M KCl, 8 M urea, and 20 mM PBS pH 7.0). Subsequently, 40 μL of a solution with or without 10 mM DTNB (5,5′-dithiolated [2-nitrobenzoic acid]) was added. The mixture was incubated in a water bath at 40 °C for 25 min. The absorbance of the resulting supernatant was measured at a wavelength of 412 nm using spectrophotometry. The total sulfhydryl content was calculated based on a molar extinction coefficient value of 13,600 M^−1^cm^−1^ and expressed as μmol/g prot. For the determination of free sulfydryl (SH) groups, a mixture consisting of 40 μL of a solution containing 10 mM DTNB and 1 mL of a cell-free extract was prepared and incubated at 25 °C for 20 min, followed by the measurement of absorbance at 412 nm (Infinite M200 Pro Tecan laboratory equipment Co., Ltd., Shanghai, China).

#### 2.5.3. 1,1-Diphenyl-2-Picrylhydrazyl (DPPH) Free Radical Scavenging Activity

Determination was carried out referring to the method of Moeun et al. [28], with slight modifications. In total, 20 μL of the sample (protein content 1.2 mg/mL) was added to 180 μL of a 0.2 nM DPPH ethanol solution. After incubation at 25 °C (water bath) in the dark for 30 min, the mixture was centrifuged at 10,000× *g* for 10 min. The absorbance was determined at 571 nm (Infinite M200 Pro Tecan laboratory equipment Co., Ltd., Shanghai, China), and the PBS (0.1 M pH 7.0) and ethanol were used as control group (A_control_) and blank group (A_Blank_), respectively.

$$\mathrm{DPPH}\;\mathrm{free}\;\mathrm{radical}\;\mathrm{scavenging}\;\mathrm{rate}\;(\%)=[1\;-(A_{\mathrm{Sample}}\;-\;A_{\mathrm{Blank}})/A_{\;\mathrm{Control}}]\times 100$$

#### 2.5.4. 2,2′-Azino-Bis(3-ethylbenzothiazoline-6-sulfonic acid) Free Radical (ABTS) Scavenging Rate

The method proposed by Zheng et al. [29] was adopted, with minor adjustments. In total, 20 μL of the sample (protein content 1.2 mg/mL) was added to 180 μL of an ABTS radical solution. After incubation at 37 °C in the dark for 6 min, the absorbance of supernate was measured at 734 nm (Infinite M200 Pro Tecan laboratory equipment Co., Ltd., Shanghai, China) after centrifugation at 10,000× *g* for 10 min. Distilled water was used to replace the sample as the control group (A_Control_), and PBS (0.05 M pH 7.0) buffer solution was used to replace the sample as the blank group (A_Blank_).

$$\mathrm{ABTS}\;\mathrm{free}\;\mathrm{radical}\;\mathrm{scavenging}\;\mathrm{rate}\;(\%)=[1-(A_{\mathrm{Sample}}\;-\;A_{\mathrm{Blank}})/A_{\;\mathrm{Control}}]\times 100$$

#### 2.5.5. Fe^2+^ Chelation Capacity

In total, 1 mL of the sample (protein content 1.2 mg/mL) was blended with 0.05 mL of 2 mM FeCl_2_ and 1.85 mL of distilled water. Then, 0.1 mL of 5 mM ferrozine was added to the mixture to initiate the reaction, and the system was maintained at room temperature for 10 min with consistent shaking. The absorbance of the mixture (A_sample_) at a wavelength of 562 nm (Infinite M200 Pro Tecan laboratory equipment Co., Ltd. Shanghai, China) was measured; the absorbance of deionized water instead of the sample (A_control_) was measured as a control.

$${\mathrm{Fe}}^{2+}\;\mathrm{chelation}\;\mathrm{capacity}\;(\%)=[A_{\;\mathrm{Control}}-A_{\;\mathrm{Sample}}/A_{\;\mathrm{Control}}]\times 100$$

#### 2.5.6. Fe^3+^ Reducing Capacity

The method for determination of the Fe^3+^ reducing capacity was slightly modified based on Shen et al.’s method [30]. In total, 2 mL of the sample (protein content 1.2 mg/mL) was mixed with a 2 mL potassium ferricyanide solution and 2 mL PBS (0.1 M pH 7.0), followed by incubation at 50 °C for 20 min. Subsequently, a 2 mL trichloroacetic acid solution was added and centrifuged at 3000 r/min for 10 min. After centrifugation, the 2 mL supernatant was mixed with 1 mL distilled water and 0.2 mL ferric trichloride solution, and the absorbance value of the mixture was measured (A_sample_) at a wavelength of 700 nm (Infinite M200 Pro Tecan laboratory equipment Co., Ltd., Shanghai, China) after being incubated for an additional 10 min at room temperature. The control group was established using PBS instead of the sample (A_Control_).

$${Fe}^{3+}\;\mathrm{reducing}\;\mathrm{capacity}\;(\%)=[1-A_{\mathrm{Control}}/A_{\mathrm{Sample}}]\times 100$$

#### 2.5.7. Hydroxyl Radical Scavenging Rate (·OH)

The method described by Quatravaux et al. [31] is referenced, with slight modifications. In total, 200 μL of the sample (protein content 1.2 mg/mL) was mixed with 200 μL of salicylic acid (5 mM, in ethanol), 200 μL of ferrous sulfate (5 mM), and 200 μL of a hydrogen peroxide solution (3 mM). The reaction was conducted at 37 °C for 30 min. After centrifugation at 3000× *g* for 5 min, the absorbance of the supernatant (A_sample_) at a wavelength of 510 nm (Infinite M200 Pro Tecan laboratory equipment Co., Ltd., Shanghai, China) was measured by replacing the sample with PBS (0.5 M pH 7.0), which was defined as the control group (A_Control_).

$$\cdot \mathrm{OH}\;\mathrm{scavenging}\;\mathrm{rate}\;(\%)=[1-A_{\mathrm{Control}}/A_{\mathrm{Sample}}]\times 100$$

#### 2.5.8. Superoxide Anion Free Radical (O_2−_) Scavenging Activity

The method of O_2−_ scavenging activity developed by Lingjiao Z et al. [32] was utilized in this present study, with minor adjustments. In total, 200 μL of the sample (protein content 1.2 mg/mL) was added to 520 μL of a 50 mM Tris-HCl solution (pH 7.5, containing 10 mM EDTA) and incubated at 37 °C for 20 min. Subsequently, 80 μL of a pyrogallotic acid (in 25 mM HCl) solution was added, and the reaction was terminated by adding 100 μL of HCl (1 M) after incubation at 37 °C for 10 min. The resulting supernatant after being centrifuged at a speed of 3000× *g* for 5 min (A_sample_) was used for absorbance determination (325 nm). The absorbance of PBS (0.1M pH 7.0 as a replacement of the sample) was also measured as a control group (A_Control_).

$$O_{2-}\;\mathrm{radical}\;\mathrm{scavenging}\;\mathrm{activity}\;(\%)=[1-A_{\mathrm{Control}}/A_{\;\mathrm{Sample}}]\times 100$$

#### 2.5.9. Anti-Peroxidation of Lipid (LP)

The method was conducted according to Ghani et al. [33], with slight modifications. The reaction mixture consisted of 0.5 mL distilled water, 1 mL 1% ferrous sulfate, and 1 mL 0.5% linoleic acid emulsion. Subsequently, 200 μL the sample (protein content 1.2 mg/mL) was added to a total volume of 500 μL reaction mixture and incubated at 37 °C in darkness for 2 h. Following this incubation period, a solution containing 40 μL of trichloroacetic acid (4%) and 400 μL of thiobarbituric acid (0.8%) was added into the reaction mixture. The resulting mixture was maintained at 100 °C (water bath) for 30 min and then centrifuged at 4000× *g* for 15 min. Finally, the absorbance of the supernatant was measured (A_sample_) at 532 nm (Infinite M200 Pro Tecan laboratory equipment Co., Ltd., Shanghai, China). Distilled water instead of the sample served as the control (A_Control_).

$$\mathrm{Inhibition}\;\mathrm{of}\;\mathrm{lipid}\;\mathrm{peroxidation}\;(\%)=[A_{\;\mathrm{Control}}-A_{\mathrm{Sample}}/A_{\mathrm{Control}}]\times 100$$

#### 2.5.10. The Systematic Assessment of Antioxidant Activity

The cell-free extract obtained by growth mentioned above was subjected to antioxidant ability detection. The total antioxidant capacity (T-AOC) and the enzyme activities of superoxide dismutase (SOD), catalase (CAT), glutathione peroxidase (GSH-Px), reactive oxygen species (ROS), reduced glutathione (GSH), and oxidized glutathione (GSSG) in the samples (100 μL intracellular cell-free extracts) were detected by T-AOC (Item No. A015-2), SOD (Item No. A001-3), CAT (Item No. A007-1), GSH-Px (Item No. A005), ROS (Item No. E004), GSH, and GSSG (Item No. A061-1) assay kits from Nanjing Jiancheng Bioengineering Institute (Nanjing, China)

### 2.6. Sodium Dodecyl Sulfate Polyacrylamide Gel Electrophoresis (SDS-PAGE)

The protein content of the cell-free extract was determined using a BCA Assay Kit (Thermo Fisher Scientific Co., Ltd., Waltham, MA, USA) with bovine serum albumin as a standard. The protein contents of the samples were adjusted to 1.2 mg/mL by PBS (0.1 M pH 7.0). SDS-PAGE was conducted according to Lee et al.’s methods [34], with a slight modification. A total 200 μL of the samples were mixed with 400 μL of a loading buffer (containing 10% SDS, 50% glycerol, 0.05%bromophenol blue, 5% β-mercaptoethanol, 1 M Tris-HCl, pH 6.8), the addition of β-mercaptoethanol was omitted by the other group, and then heated at 60 °C for 30 min. A 40 μL of the cell-free extract was loaded for the electrophoresis that was performed by a discontinuous SDS-PAGE system with a 4% acrylamide stacking gel and a 12.5% separating gel.

The gels were mounted in a vertical slab gel apparatus (Mini-PROTEAN Tetra Cell, Bio-Rad Laboratories, Inc., Hercules CA, USA) using a basic power supply (Bio Rad mini, Bio-Rad Laboratories, Inc., Hercules, CA, USA). Electrophoresis was performed at a constant 90 V for 1.5 h. The protein bands in the gel were stained by Coomassie Brilliant Blue R-250 for 1 h and then distained by the ddH_2_O over night (the water was changed every 2 h). The gels were scanned using the Bio-Rad imaging system (ChemiDocTM XRS+, Bio-Rad Laboratories, Inc., Hercules CA, USA). The gels were analyzed using ImageJ.

### 2.7. Determination of Quality Indicators for Lactic Acid Beverages

Skim milk powder (Fonterra Trading Co., Ltd., Shanghai, China), sucrose (Swire Sugar Co., Ltd., Beijing, China), and sucrose (Ubojia Food Co., Ltd., Nanchang, China.) were thoroughly mixed in water at a temperature of 70 °C. The mixture was then filtered through a 200 mesh sieve and subjected to homogenization at a pressure of 200 bar (SPX high pressure homogenizer, Speck Fluid Technology Co., Ltd., Shanghai, China) for two cycles. Subsequently, the resulting mixture was autoclaved at a temperature of 115 °C for 15 min (SX-500 autoclave, Tomy Digital Biotechnology Co., Ltd., Hercules, CA, USA) and cooled down to 37 °C before use. The cooled milk matrix was inoculated with the twice activated NJAU-01 (2.5%, *v*/*v*) strain and incubated at a temperature of 37 °C to obtain the NJAU-01 lactic acid beverage.

In the subsequent experiments, Y, a commercially available lactic acid beverage, similar to the NJAU-01 lactic acid beverage formulation, was used as the positive control in comparison to the NJAU-01 lactic acid beverage. The symbols A0 and Y0 represented the NJAU-01 lactic acid beverage and Y lactic acid beverage stored for 0 days, respectively. Similarly, AM and YM represented the NJAU-01 lactic acid beverage and Y lactic acid beverage stored for 21 days, respectively.

The antioxidant indices of the lactic acid beverages were also assessed using the aforementioned methods in Section 2.2, Section 2.5.1, Section 2.5.4, Section 2.5.5 and Section 2.5.7. The experimental subjects were lactic acid beverages stored for 0, 7, 14, and 21 days.

#### 2.7.1. Sensory Evaluation

Ten food science students (5 males and 5 females) who had undergone a Descriptive Analysis Test, a standardized qualitative method employed for sensory evaluation to identify and quantify the sensory attributes of a product, were selected to constitute a panel for conducting sensory evaluations. The samples were evaluated sequentially based on their color, aroma, flavor, and texture (Table S1). Furthermore, this study included a comprehensive statistical analysis of the results obtained from the sensory test. The data were analyzed using analysis of variance (ANOVA) to determine if there were any significant differences in the sensory attributes among the different samples.

#### 2.7.2. Response Surface Methodology (RSM)

Building upon the results of previous single-factor experiments, in conjunction with the response surface design scheme and the comprehensive fuzzy mathematical model, inoculum, fermentation time, and sugar addition were identified as the three critical factors. Sensory evaluation and total antioxidant capacity (T-AOC) were utilized as response variables. The refined NJAU-01 lactic acid beverage will be utilized for subsequent experimental assessments.

#### 2.7.3. The Count of Viable Bacteria

The viable bacterial count was determined using the pour plate method. Following agitation, 1 mL of a lactic acid bacteria beverage was transferred into 9 mL of sterile Ringer’s solution (0.9% NaCl *w*/*v*), and subsequent gradient dilutions were performed. The appropriate dilution (10^−6^) was then cultured on sterile Petri dishes containing MRS solid medium at 37 °C for 48 h, and plates with a total colony count ranging from 30 to 300 were selected for enumeration. The results are expressed as log CFU/mL (CFU, colony-forming units).

#### 2.7.4. Titratable Acidity

Measure 10 g of the sample in a conical flask, add 20 mL of pre-boiled water cooled to room temperature, dissolve the sample completely, stir well, introduce 2 mL of phenolphthalein indicator solution, mix thoroughly, and titrate with a standardized solution of 6 M sodium hydroxide until the color matches that of the reference solution and remains stable for at least 5 s. Throughout the titration process, ensure continuous stirring using a magnetic stir bar while blowing nitrogen into the conical flask to prevent carbon dioxide absorption from ambient air. Record the volume (V_1_) of sodium hydroxide solution used accurately to within 0.05 mL and substitute it into the calculation equation. For blank titration purposes, use distilled water as a control experiment with a volume (V_0_) measurement taken for the consumption of a sodium hydroxide standard solution.

$$Titratable\;acidity\left({}^{\circ}T\right)=\frac{6\;\times \;\left(V_{1}\;-\;V_{0}\right)\times 100}{10\;\times \;0.1}$$

6—The concentration of the sodium hydroxide standard solution (mol/L);
V_1_—The volume of the sodium hydroxide standard solution consumed during titration (mL);
V_0_—The volume of the sodium hydroxide standard solution consumed during blank titration (mL);
100—100 g of the sample;
10—The mass of the sample taken (g);
0.1—The molar concentration of sodium hydroxide defined by the acidity theory (mol/L).

#### 2.7.5. Determination of Viscosity

The viscosity was determined using a viscometer (NDJ-8S Fangrui Instrument Co., Ltd., Shanghai, China), and the samples were equilibrated at 25 °C for 10 min prior to determination, employing a No. 1 rotor operating at 36% torque and a speed of 60 revolutions per minute.

#### 2.7.6. Centrifugal Sedimentation Rate

The method proposed by Zhao et al. [35] was slightly modified for the determination of lactic acid beverage stability. To determine the stability, 10 mL of a milk beverage was transferred into a centrifuge tube and subjected to centrifugation at 3000× *g* for 10 min at 4 °C. After standing for 5 min, the supernatant was discarded. The ratio between the mass of the precipitate obtained from centrifugation and the initial sample mass represented the centrifugal precipitation rate.

#### 2.7.7. Determination of Flavor Substances (GC-MS)

The samples were chosen from beverages stored for 0 and 21 days. For sample handling, refer to Lee et al. [36], with minor adjustments. In total, 10 mL of a lactic acid beverage sample was placed in a 50 mL headspace extraction bottle, followed by the addition of 4 mL o-dichlorobenzene as an internal standard. The bottle was then sealed and refrigerated at 4 °C. During the extraction process, the samples were pre-incubated for 10 min, extracted for 30 min, and desorbed at 220 °C for 3 min. The test conditions were as follows: TR-FFAP column was utilized with carrier gas helium (99.999% purity) flowing at a rate of 1 mL/min in non-split mode. The injection port temperature was set at 230 °C initially, held for three minutes before being raised to a temperature of 140 °C at a rate of increase of 5 °C/min and maintained for another three minutes, subsequently increased to a final temperature of 220 °C at a rate of 7 °C/min, and maintained again for 3 min before undergoing desorption for an additional 3 min. Mass spectrometry conditions included an EI ion source operating at 70 ev with an ion source temperature set to 250 min and mass scan range spanning from 33 to 450 amu. Qualitative and quantitative analysis involved using PerkinElmer TurboMass Ver6.1.2 software, which automatically searched the NIST2017 spectrum library; substances exhibiting positive or negative matching degrees ≥ 700 were screened accordingly. The content of flavor substances in each group was calculated using O-dichlorobenzene as the reference internal standard.

#### 2.7.8. Odor Activity Value (OAV)

The odor activity value (OAV) is a quantitative measure of the volatile compound’s contribution to the overall odor perception in a specific sample. This value is determined by comparing the concentration of the compound present in the sample with its olfactory threshold [37,38,39,40], which represents the minimum detectable concentration by the human olfactory system.

### 2.8. Statistical Analysis

All analytical tests were carried out in triplicate. The results were statistically analyzed by one-way ANOVA, and comparison of the mean values was performed by Duncan’s test using SPSS 21. Principal component analysis (PCA) was performed using the statistical software SIMCA 14.1 (MKS Instruments AB Inc., Malmo, Sweden). The MDA scavenging ability, oxidative stress indices, and antioxidant parameters of NJAU-01 at various growth stages were considered as variables in the NJAU-01 experiment. The types and contents of all volatile compounds were utilized as variables in the beverage experiments. The plots were generated using Origin 2021 pro. Significance was considered at *p* < 0.05.

## 3. Results and Discussions

### 3.1. Oxidation Indices of NJAU-01 Under 40 mM MDA Stress

**Figure 1:**
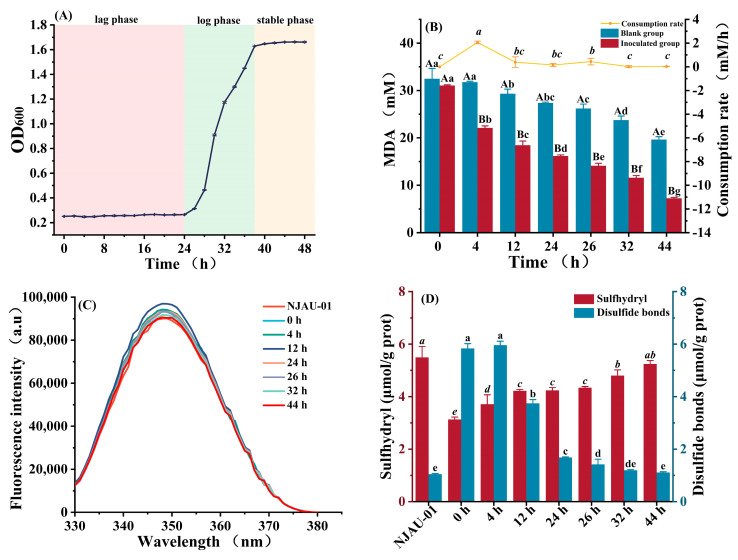
Growth curve of *L. plantarum* NJAU-01 in 300 mL MRS culture system with 40 mM MDA (**A**). Malondialdehyde content and consumption rate during kinetic growth of *L. plantarum* NJAU-01 (**B**). Schiff base content of *L. plantarum* NJAU-01 cell-free extract (**C**). Total thiol and disulfide bond content of *L. plantarum* NJAU-01 cell-free extract (**D**).

Malondialdehyde (MDA), formed by the reaction of polyunsaturated fatty acids and free radicals, leading to lipid peroxidation of cellular membranes, is widely recognized as a key biomarker for oxidative stress [41]. The presence of MDA subsequently leads to the formation of peroxyl radical dimers or alkoxyl radicals, ultimately resulting in cell damage [42]. Our results showed that NJAU-01 exhibited optimal resistance to MDA at a concentration of 40 mM (Figure S1), beyond which (at 45 mM MDA concentration) it entered a lethal state. The duration of both lag and logarithmic phases of NJAU-01 was prolonged after being exposed to 40 mM MDA (Figure 1A), and NJAU-01 in MRS (containing 40 mM MDA) showed a significant decrease throughout the incubation period (*p* < 0.05). Under MDA oxidative stress, NJAU-01 was able to maintain the integrity of cellular membranes and mitigate the impact of oxidative stress through defense and repair systems, which allowed the cells to utilize nutrients for 24 h growth, reaching a stable growth phase at 44 h. Notably, the consumption rate (2.06 mM/h) of MDA at 4 h was significantly (*p* < 0.05) higher compared to the other time points (Figure 1B). Our findings were consistent with the results of Basu et al. [43], where 0.96 mM hydrogen peroxide similarly induced changes in the growth state of the strain. In the CK group, the concentration of MDA gradually decreased with the extension of the culture time, which might be attributed to the inherent instability of MDA in the solution. In an aqueous solution at room temperature and near-neutral pH, MDA is prone to undergo self-condensation of aldehydes and reversible hydrolysis, resulting in the formation of dimers, oligomers, acetaldehyde, and formic acid [44]. Some studies have indicated that approximately 68% of MDA undergoes aldol self-condensation reactions after 24 h at a relatively high temperature (40 °C) [45].

However, during the lag phase, the MDA concentration decreased significantly in the presence of NJAU-01compared to the CK group. This phenomenon could be attributed to the interactions between MDA molecules and specific bacterial protein groups, such as those containing lysine, cysteine, or tyrosine residues, which facilitate the formation of cross-links between proteins, leading to the generation of substances like Schiff bases and disulfide bonds [46]. The fluorescence intensity of the Schiff base in cell-free extracts (Figure 1C) demonstrated a prominent absorption peak near a scanning wavelength of 350 nm, with its highest level observed at 12 h, followed by a subsequent decline as the culture time extended. As depicted in (Figure 1D), the initial sulfhydryl content decreased (to 3.13 μmol/g prot at 0 h), and then increased over time (*p* < 0.05). The disulfide bond content initially increased, followed by a subsequent decrease (*p* < 0.05), which reached a peak of 5.96 μmol/g prot at 4 h. During the lag phase, MDA likely contributed to the binding of active groups on the protein surface, decreasing total sulfhydryl content and increasing disulfide bond formation. By the stationary phase (44 h), the total sulfhydryl content increased significantly (*p* < 0.05), indicating that bacteria alleviated MDA oxidation and underwent vigorous growth and reproduction, exposing more protein surface groups and reducing the level of protein oxidation. This finding aligns with the results of Zhang et al. [47]. Wu et al. [48] also discovered in their study that MDA, a product of rice bran rancidity caused by storage, led to a decrease in free sulfhydryl group content and an increase in disulfide bond content. The free amino groups of proteins (lysine, arginine, glutamine side chains, and N-terminal amino groups) can react with aldehydes generated by lipid peroxidation to form Schiff bases [49], which in turn facilitate protein cross-linking and thereby alter the protein structure [50]. The appearance of fluorescence peaks may be attributed to the reaction between MDA and proteins. Simultaneously, the MDA-oxidized protein cross-linking resulted in a decrease in total sulfhydryl content and an increase in disulfide bond content, which is consistent with previous results of this study.

Blank group: This group was subjected to the MRS+MDA sterile system. Inoculated group: This group was exposed to the MRS+MDA+NJAU-01 system. NJAU-01: *L. plantarum* NJAU-01 under non-MDA stress. Different capital letters indicate significant (*p* < 0.05) differences in MDA concentration among different groups at the same time point. Different lowercase letters denote statistically significant variations (*p* < 0.05) at different time points within the same group.

### 3.2. Changes in Antioxidant Activity and Antioxidant Enzyme Activity In Vitro

**Figure 2:**
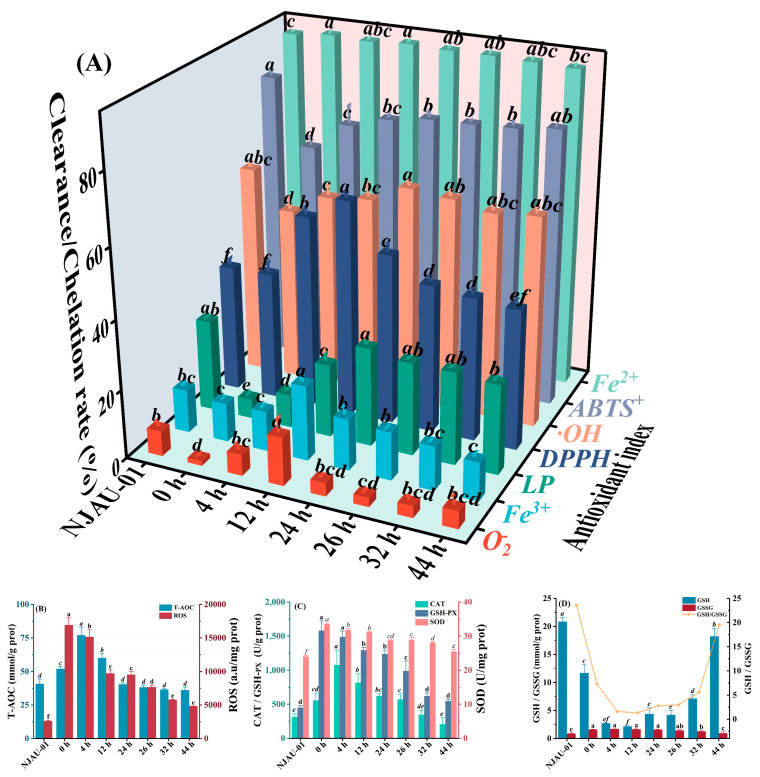
Changes in antioxidant indicators (O_2−_, Fe^3+^, LP, DPPH, OH, ABTS+, Fe^2+^) in vitro (**A**); T-AOC and ROS content (**B**); CAT, GSH-Px, and SOD activity (**C**); GSH, GSSG activity, and the ratio of CSH and GSSG of cell-free extract of *L. plantarum* NJAU-01 in 300 mL MRS culture system with 40 mM MDA (**D**). T-AOC: total antioxidant capacity; ROS: reactive oxygen species; CAT: catalase; SOD: superoxide dismutase; GSH-Px: glutathione peroxidase; GSH: reduced glutathione; GSSG: oxidized glutathione. NJAU-01: L. plantarum NJAU-01 under non-MDA stress. The different lowercase letters represent significant (*p* < 0.05) variations in each antioxidant activity between different time points.

Under 40 mM of MDA stress, the antioxidative activities of NJAU-01 varied across different growth stages (Figure 2A). The O_2−_ scavenging activity, Fe^3+^ reducing capacity, DPPH radical scavenging activity, and Fe^2+^ chelating ability significantly increased (*p* < 0.05) before 12 h, reaching their maximum values at 13.97%, 21.70%, 62.23%, and 93.08%, respectively. Similarly, the capacity to inhibit lipid peroxidation and ·OH scavenging initially exhibited a significant increase followed by a subsequent decrease (*p* < 0.05) during the growth stage of NJAU-01, peaking at 24 h with values of 28.42% and 62.67%, respectively. However, the ABTS^+^ scavenging rate demonstrated a continued increase throughout the growth stage, reaching its maximum value of 79.79% at 44 h.

The formation of structural disulfide bonds in proteins can also be catalyzed by enzymes such as oxidoreductases and thiol oxidases, and the presence of ROS can directly or indirectly induce alterations in sulfhydryl groups [51].

The intracellular ROS content is a direct indicator of cellular oxidative stress, which induced the oxidation of macromolecules in cells, damaged cell membranes, altered the activity of cytoplasmic enzymes, affected growth and metabolism, and hindered the functional characteristics of lactic acid bacteria [52,53]. The fluorescence intensity of ROS was highest at 0 h and significantly (*p* < 0.05) decreased during the lag phase (Figure 2B). This likely resulted from the high concentration of MDA, which exposed the bacterial cells to severe strong oxidative stress, thereby causing cellular damage and a sharp increase in intracellular ROS levels. As NJAU-01 entered the lag phase, it gradually alleviated MDA oxidation over time. This mitigation allowed the bacteria to utilize the nutrients in the culture system for growth and potentially secreted antioxidant substances, leading to a significant reduction in ROS intensity. This observation aligns with the discovery made by Zhang et al. [54], who reported that oxidative stressors such as nitrogen deficiency, phosphorus deficiency, strong light, and high salinity led to lipid accumulation and increased ROS content in Chlorella vulgaris.

The antioxidant capacity (T-AOC) of NJAU-01 initially increased, reaching a maximum value of 77.02 mmol/g prot at 4 h, followed by a significant decrease (*p* < 0.05). The activities of catalase (CAT), glutathione peroxidase (GSH-Px), and superoxide dismutase (SOD) in NJAU-01 all exhibited an increase and followed with a subsequent decrease under 40 mM MDA stress (Figure 2C). Specifically, CAT activity peaked at 4 h with a value of 1076.47 U/g prot, while GSH-Px and SOD enzymatic activities attained their maximum levels at 0 h under stress, measuring 1578.00 U/g prot and 33.61 U/mg prot, respectively. Kang et al. [55] designed an intestinal mucosal epithelial cell model and through immunofluorescence analysis, observed a significant increase in ROS fluorescence intensity in the deoxynivalenol-induced cell model. This increase was accompanied by the enzymatic activities of CAT and GSH-Px significantly decreasing, causing apoptosis and inflammation in intestinal epithelial cells. GSH can serve as a substrate to bind to GSH-Px and GST, facilitating the decomposition of hydroperoxides, protecting cells from oxidative damage and exogenous toxins, and maintaining redox homeostasis [56]. Figure 2D illustrates a significant initial decrease followed by a subsequent increase in GSH levels, reaching the lowest point of 2.16 mmol/g prot at 12 h (*p* < 0.05). Conversely, the content of GSSG increased, which was then followed by a subsequent decrease, and peaked at a maximum of 1.69 mmol/g prot at 4 h. The GSH content significantly decreased during the lag phase, coinciding with an increase in intracellular ROS fluorescence intensity (Figure 2B). These findings indicated that MDA oxidation at high concentrations induced a bacterial response, leading to a sharp decline in antioxidant capacity. However, after the lag phase, GSH content rose notably, suggesting a gradual strengthening of bacterial antioxidant defenses. The decrease in intracellular ROS fluorescence intensity can be attributed to the clearance of hydroperoxides and other oxidative stress products, which corroborates these findings. Some studies have shown that alleviating oxidative stress by modifying and activating uncoupling proteins leads to a reduction in mitochondrial ROS and the activation of antioxidant response pathways, including an increase in GSH levels [57]. Moreover, under oxidative conditions, GSH readily forms mixed disulfides with protein cysteines [56], resulting in a decrease in GSH content and an increase in disulfide bond content (Figure 1D).

### 3.3. SDS-PAGE

**Figure 3:**
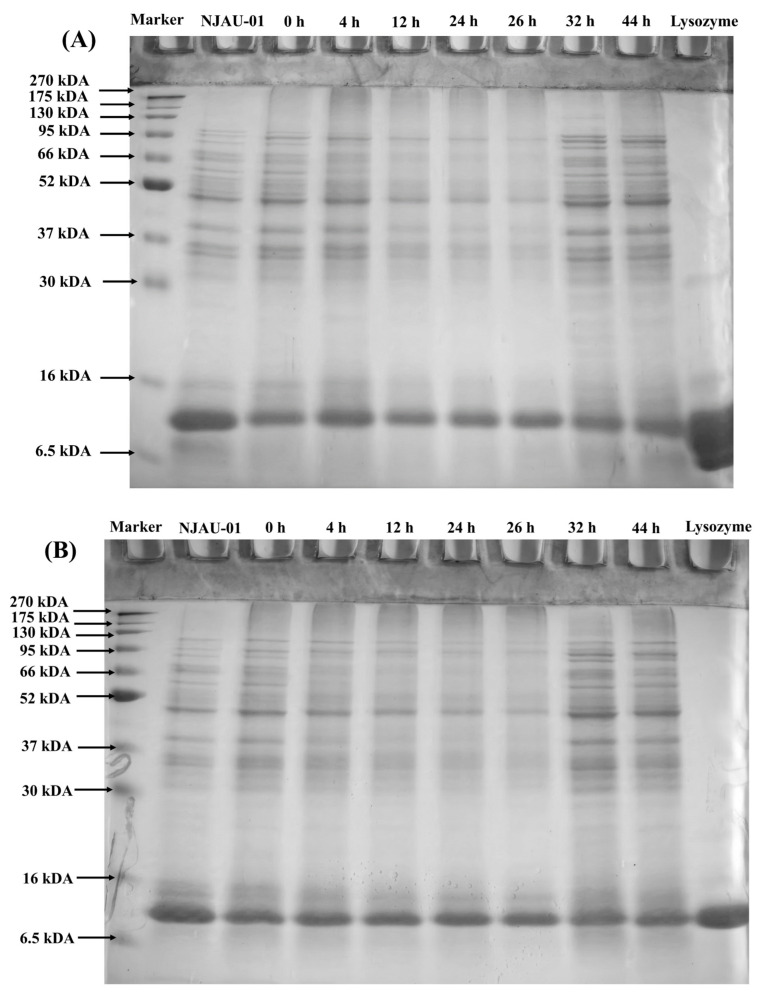
SDS-PAGE analysis of cell-free extracts from *Lactobacillus plantarum* NJAU-01 at different growth stages under 40 mM MDA with presence of mercaptoethanol (**A**), and without the presence of mercaptoethanol (**B**).

SDS-PAGE analysis demonstrated variations in the intensity of protein bands within cell-free extracts of NJAU-01 across different growth stages (Figure 3). Protein content notably decreased during incubation periods ranging from 12 h to 26 h. A high molecular weight polymer was observed at the top of the gel (270 to 130 kDA), reaching peak intensity at 4 h (Table 1) and then gradually diminishing and disappearing as incubation time extended. These observations suggested that NJAU-01 underwent dynamic protein upregulation and downregulation as they adapted to mitigate MDA stress. By comparing Figure 3A and Figure 3B, it is evident that an aggregation occurs in the band above 130 kDA; the group lacking mercaptoethanol exhibited a higher band intensity compared to the group containing mercaptoethanol. This can potentially be attributed to cross-linking induced by an increase in disulfide bonds. This observation aligns with the findings reported by Chen et al. [15].

### 3.4. Principal Component Analysis (PCA)

**Figure 4:**
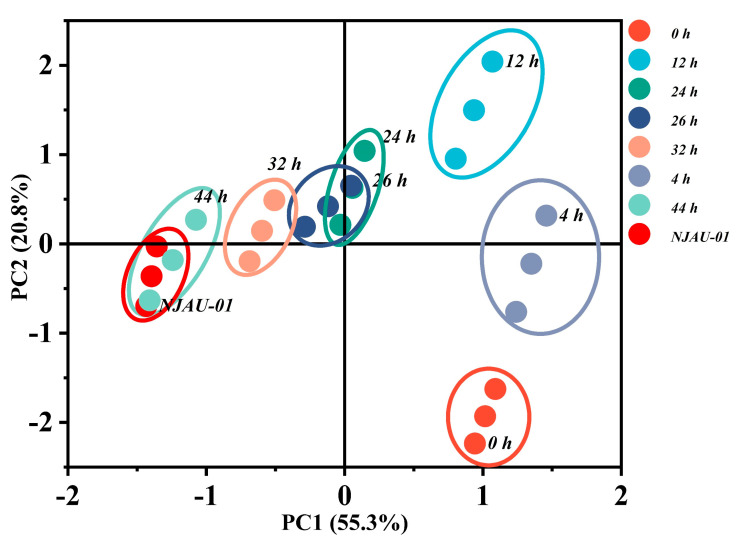
Principal component analysis (PCA) of *Lactobacillus plantarum* NJAU-01 under 40 mM MDA stress at different growth stages.

The two main components accounted for 55.3% and 20.8% of total variation in PCA, respectively (Figure 4). The samples of 44 h and non-MDA treated NJAU-01 were located on the same side of the vertical axis, which are clearly separated from the other samples. Moreover, the samples at 0 h, 4 h, 12 h, 24 h, 26 h, and 32 h were distributed in the first, second, and third quadrants, respectively. The responses of MDA-stressed NJAU-01 on protein oxidation, in vitro antioxidant capacity, and antioxidant enzyme activity indicators throughout the growth stage were presented as a circular rotation on the PCA plot.

### 3.5. Response Surface

The results, with sensory scores and total antioxidant capacity as response variables, are presented in (Table 2A).

The multivariate quadratic regression simulation equation obtained a sensory score as the response value.

Sensory score = 83.72 + 9.72A − 0.89B + 5.35C − 2.1AB + 2.5AC + 3.19BC − 6.04A^2^ − 6.60B^2^ − 7.16C^2^.

The multivariate quadratic simulation equation obtained T-AOC as the response value.

T-AOC = 7.15 + 0.32A + 0.098B + 6.125 × 10^−3^C + 0.030AB + 1.000 × 10^−3^AC + 0.044BC + 0.026A^2^ − 0.39B^2^ − 0.10C^2^.

A—Inoculum;

B—Fermentation time;

C—Sugar addition.

#### 3.5.1. Effect of Three-Factor Interaction on Sensory Evaluation

The analysis of variance for the quadratic model of the response surface revealed that the *p*-value is <0.0001, the Lack of Fit is 0.2262 > 0.05, and R^2^ is 0.9905 > 0.9, suggesting that the quadratic regression model effectively predicted response values with minimal interference from other factors (Table 2B). Based on the F values (Table 2B), the factors influencing sensory evaluation were ranked as follows: A (Inoculum) > C (Sugar addition) > B (Fermentation time). Among these factors, the *p*-value of A and C was <0.0001, indicating a highly significant impact on the sensory qualities of lactic acid beverages. However, the *p*-value of B was 0.1418 (>0.05), suggesting that it had no significant impact on the sensory qualities.

**Figure 5:**
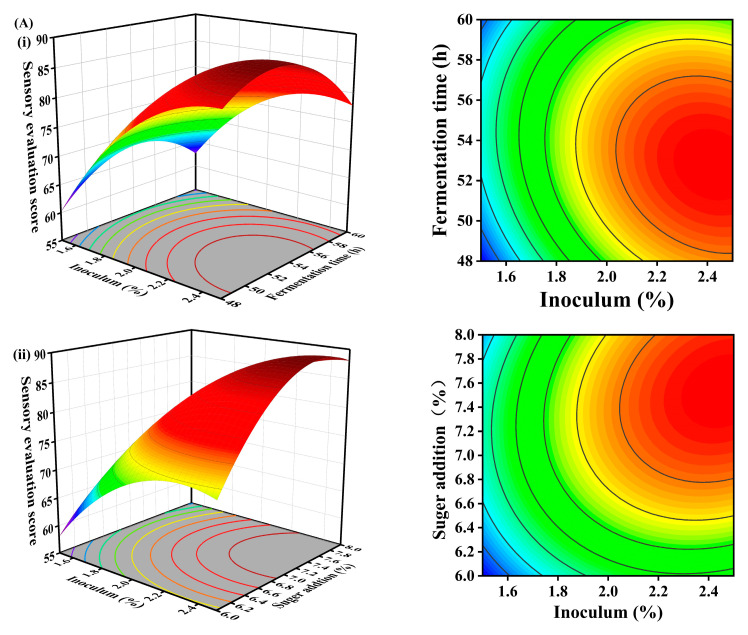

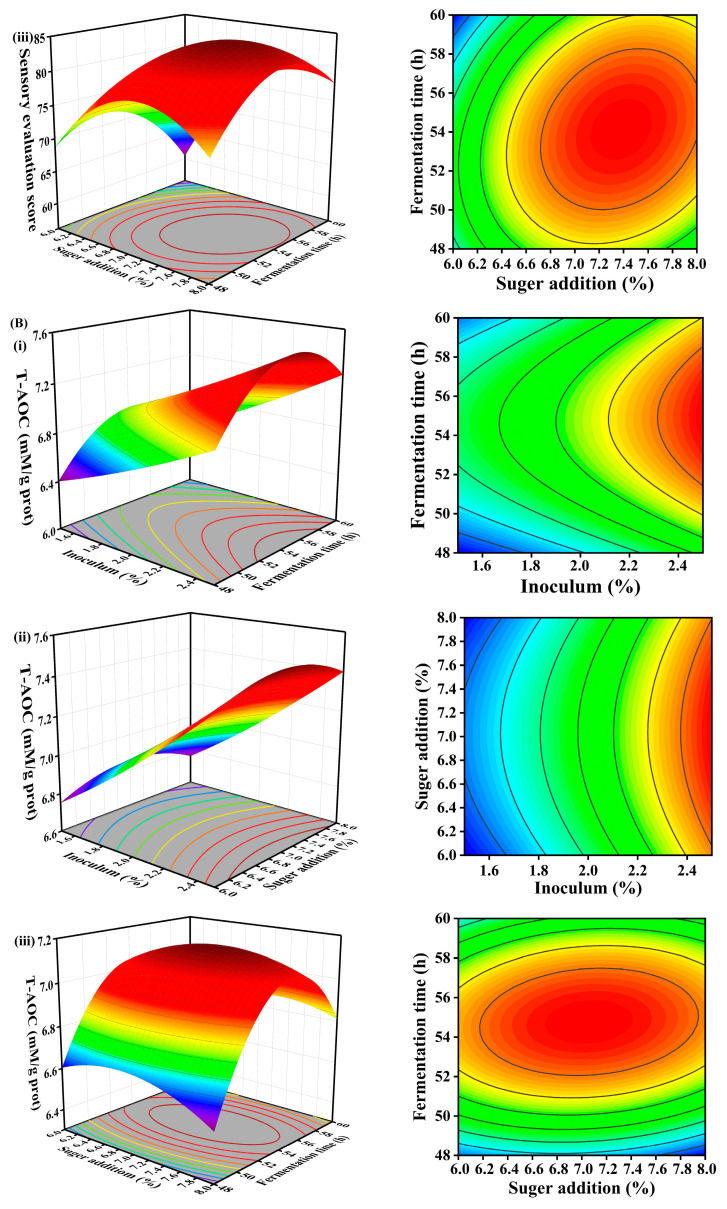
(**A**): The influence of the interaction among inoculum, fermentation duration, and sugar addition on the sensory evaluations of NJAU-01 lactic acid beverages. (**B**): The impact of the interaction among inoculum, fermentation duration, and sugar addition on total antioxidant capacity (T-AOC) in the NJAU-01 lactic acid beverage. (**i**) Represents the interaction between the inoculation amount and fermentation time. (**ii**) Denotes the interaction between the inoculation amount and sugar addition. (**iii**) Signifies the interaction between fermentation time and the sugar addition. As the color approaches red, the value increases; conversely, as the color shifts towards blue, the value decreases.

Furthermore, a highly significant interaction was observed between B and C factors (*p*-value = 0.0041). Significant interactions were also found between A/B factors and A/C factors (*p*-values < 0.05). Moreover, the squared terms A^2^, B^2^, and C^2^ exhibited a lower *p*-value (<0.001), further highlighting their significance in sensory evaluations. The 3D response surface and the 2D contour plot illustrated the influence of the interaction between the two factors on the sensory attributes of lactic acid beverages (Figure 5A). A flatter 3D response surface indicated a less significant influence of the factor, whereas a more curved surface suggested a more pronounced effect. Additionally, contour lines that formed closer to a circular shape signified a weaker interaction between these factors.

#### 3.5.2. Effect of Three-Factor Interaction on T-AOC

The analysis of variance for the quadratic model of the response surface (Table 2C) revealed a *p*-value of 0.0002 (<0.001), a Lack of Fit of 0.0702 (>0.05), and R^2^ of 0.9680 (>0.9). These results suggested that the quadratic regression model effectively predicted the response value while minimizing interference from other factors. Based on the F values (Table 2C), the factors influencing T-AOC were ranked as follows: A (Inoculum) > B (Fermentation time) > C (Sugar addition). The *p*-value for factor A and B was <0.05, indicating a significant effect. Whereas the *p*-value for factor C was (0.8475) > 0.05, indicating no significant effect. Furthermore, A/B, A/C, and B/C all showed a *p*-value > 0.05, implying no significant effect on T-AOC. On the other hand, B^2^ (*p*-value < 0.001) showed a highly significant effect, while A^2^ and C^2^ (*p*-value > 0.05) indicated no significant impact from these quadratic terms.

The 3D response surface and 2D contour plot illustrated the impact of the interaction between two factors on the T-AOC of lactic acid beverages (Figure 5B). The smoothness of the 3D response surface suggested that the factor had no significant effect. The proximity of the contour lines to the circle form reflected the level of significance in their interaction. When both the sensory score and T-AOC reached their peak values, the optimal theoretical parameters were an inoculum concentration of 2.50%, a fermentation duration of 54.74 h, and a sugar addition rate of 7.28%. Based on these values, the actual production parameters were adjusted to an inoculum concentration of 2.50%, after a mentation duration of 55.00 h, and a sugar addition rate of 7.00%. The predicted actual sensory score was 86.72, while the T-AOC measured at 7.50 mM/g protein. The lactic acid beverage was prepared according to these optimized production conditions, resulting in a sensory score of 87.45 and a T-AOC value of 7.40 mM/g protein.

### 3.6. The Quality and Functions of Lactic Acid Beverages Throughout the Storage Period

**Figure 6:**
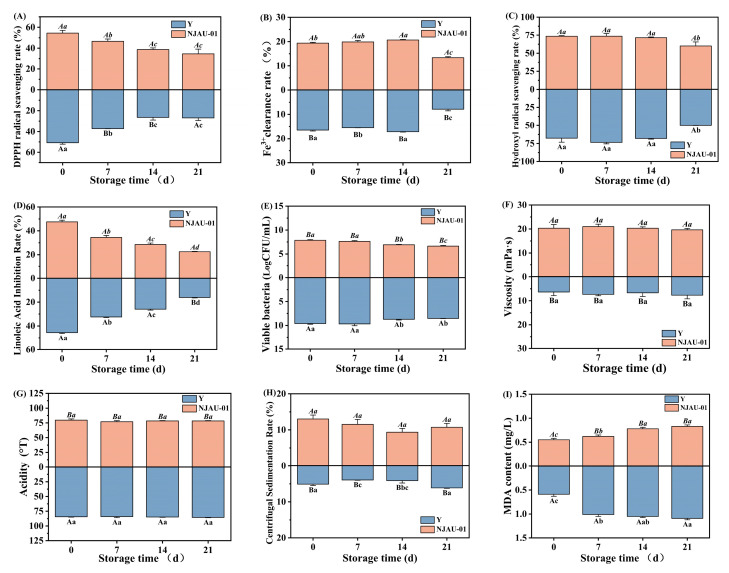
The DPPH free radical scavenging rate (**A**), Fe^3+^ reducing power (**B**), hydroxyl free radical scavenging rate (**C**), linoleic acid inhibition rate (**D**), viable bacterial count (**E**), viscosity (**F**), acidity (**G**), centrifugal precipitation rate (**H**), and MDA content (**I**) of the lactic acid beverages were evaluated throughout the storage period. The different uppercase letters indicate significant (*p* < 0.05) differences in the quality indices among the different groups simultaneously, while the different lowercase letters represent significant (*p* < 0.05) differences in the quality indices at different time points. The NJAU-01 beverage refers to the lactic acid beverage prepared by NJAU-01, while the Y beverage denotes Y lactic acid beverage products.

It is essential to discuss the quality indicators of the NJAU-01 lactic acid beverage and the Y lactic acid beverage over the 21-day storage period. A consistent and significant decline (*p* < 0.05) was observed in DPPH scavenging activity (Figure 6A), Fe^3+^ reducing capacity (Figure 6B), ·OH scavenging capacity (Figure 6C), linoleic acid inhibition rate (Figure 6D), viable bacterial count (Figure 6E), and viscosity (Figure 6F) throughout the storage period, and these antioxidant activities of the beverages is highly dependent on the fermentation strain. The following are the minimum values for these parameters that were reached at day 21: NJAU-01 (34.56%, 13.37%, 60.10%, 22.44%, 4.40 × 10^6^ and 19.67 mPa·s); and Y (26.90%, 7.89%, 50.10%, 16.07%, 3.40 × 10^8^ and 7.67 mPa·s). The NJAU-01 beverage demonstrated excellent antioxidant capacity during the storage. This might be attributed to the strain’s strong inherent antioxidant abilities and the release of bioactive peptides with antioxidant activity during the fermentation [58] thereby enhancing the antioxidant capacity of the beverage. Several studies have reported similar findings; Peng et al. [8] observed that Lactobacillus delbrueckii subsp. bulgaricus VHProbi C08 enhanced the DPPH scavenging rate and reduced lipid peroxidation in fermented soy milk. Wang et al. [59] showed that the fermentation with Lactobacillus plantarum Y16 significantly improved the antioxidant properties of fermented soybean milk, including an increased iron ion reducing power and the DPPH scavenging rate. Liu et al. [58] indicated that the addition of Lactobacillus rhamnosus 6134 enhanced the DPPH radical scavenging activity of cheddar cheese.

The inhibition rate of linoleic acid in the NJAU-01 beverage decreased from 47.54% to 22.43%, while the inhibition rate of linoleic acid in Y decreased from 45.60% to 16.07%. (Figure 6D). Shori et al. [60] analyzed cashew yogurt prepared with *Lactobacillus plantarum* LP, which also showed high iron reduction power and ferrous ion chelating capacity after 21 days of storage, but the sensory score was slightly lower than that of yogurt fermented with *Lactobacillus rhamnosus* and *Lactobacillus casei.* These results align with the outcomes of our research.

The acidity and centrifugal precipitation rate exhibited a non-significant trend of an initial decrease followed by an increase (Figure 6G,H). NJAU-01 exhibited significantly lower acidity compared to Y, while demonstrating a significantly higher centrifugal precipitation rate. Both the beverages exhibited a significant increase (*p* < 0.05) in MDA (Figure 6I) content during storage, while the MDA content of NJAU-01 (0.83 mg/L) demonstrated a significantly lower level compared to that of Y (1.09 mg/L) after 21 days of storage. Fan et al. [61] enhanced the antioxidant capacity of yogurt by adding probiotics Lactobacillus brevis 54 (with high gamma-aminobutyric acid production capacity) and Lactobacillus casei 56 (with selenium-enrichment capacity). The lower acidity in the NJAU-01 lactic acid beverage compared to the control group could be attributed to the strain’s lower acid-producing ability compared to the Y beverage. Additionally, the initial sugar concentration in the raw materials will also affect the amount of lactic acid that can be produced during fermentation, further resulting in different acidity in the beverages. Post-acidification refers to the accumulation of acid during storage [62]. The acidity of the both lactic acid beverages remained relatively stable throughout storage, indicating that there was minimal post-acidification; this stability in acidity contributed to the overall quality consistency of the product.

It is crucial to ensure the minimum probiotic levels in dairy beverages are maintained during storage [63]. In the case of NJAU-01, the viable count in the lactic acid beverage exceeded 10^6^ CFU/mL throughout the storage period, although it gradually decreased at 4 °C (Figure 6E). This is likely due to the fact that the optimal growth temperature of NJAU-01 is 37 ± 2 °C. Under the condition of 4 °C, the growth rate of NJAU-01 is slow. NJAU-01 decomposes lactose to produce lactic acid, leading to a beverage acidity of 77 to 80 °T during storage. The lower acidity of the NJAU-01 beverage may lead to a decline in bacterial survival.

### 3.7. GC-MS

#### 3.7.1. Volatile Compound Profiles

**Figure 7:**
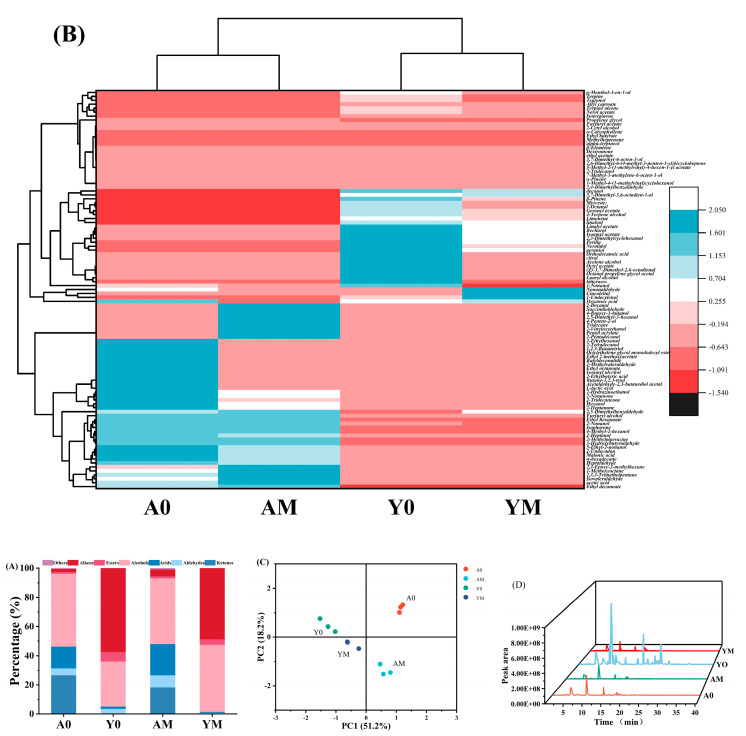
The ratio of the content of flavor substances (**A**), a clustering heat map of flavor substances (**B**), principal component analysis of all flavor substances (**C**), and the total ion chromatograms of the lactic acid beverages during storage (**D**). The symbols A0 and Y0 represent the NJAU-01 lactic acid beverage and the Y lactic acid beverage stored for 0 days, respectively. Similarly, AM and YM represent the NJAU-01 lactic acid beverage and the Y lactic acid beverage stored for 21 days, respectively.

The characteristic volatile compounds contributing to the distinct dairy aroma in lactic acid beverages can be classified as fatty acids, esters, ketones, hydrocarbons, aldehydes, esters, etc., most of which are synthesized through reactions involving or related to milk fat [64]. The primary flavor compounds in the NJAU-01 beverage comprised alcohols, acids, ketones, and aldehydes, with a minor presence of alkanes, alkenes, and esters (Figure 7A). In contrast, the Y beverage predominantly contained alkanes, alkenes, alcohols, and esters, along with lesser quantities of acids and aldehydes. During the storage period, the concentrations of acids, alkanes, alkenes, and aldehydes in the NJAU-01 beverage demonstrated an increasing trend, whereas the concentration of ketones decreased. In contrast, for the Y beverage, the alcohol content increased, while the levels of alkanes, alkenes, esters, and aldehydes decreased.

A cluster heat map of volatile flavor compounds for both lactic acid beverages NJAU-01 and Y, generated during storage (Figure 7B), demonstrated distinct clustering patterns at both the early and late stages of storage. A total of 48 and 40 volatile compounds were identified in NJAU-01 and Y, respectively. The data were normalized based on the content of the flavor compounds, indicating each beverage possessed unique volatile profiles. The samples collected at different time points were grouped into four categories. NJAU-01 beverage exhibited higher concentrations of ketones (2-nonone, heptanone, undecanone), esters (methoxy ethyl acetate, butyric decanolide, ethyl octanoate, dodecyl ester), aldehydes (acetaldehyde-2, 3-butanediol acetal, 2-methylpental), and alcohols (tetroxide, butantriol, isoamyl alcohol, n-hexanol) compared to the Y beverage on day 0 of storage. Conversely, the contents of alcohols (geraniol, nerol nonanol, fenchol, cyclohexanol perillyl alcohol), aldehydes (octanal malondialdehyde acetal, octandienal, citral perillyl aldehyde) and esters (octanoacetate, linaloacetate isoamyl acetate) in the Y beverage were higher than those in the NJAU-01 lactic acid beverage for the same storage time. The cluster analysis of the NJAU-01 beverage and Y beverage revealed that the primary distinction between these two beverages lies in their flavor substances, which can be attributed to variations in fermentation strains used. At the end of storage, the flavor profiles of the two beverages converged.

The results of the principal component analysis revealed significant variations in the flavor compounds between the two beverages during storage (Figure 7C). The flavor compounds in the Y beverage were predominantly located in the second and third quadrants, while those in the NJAU-01 beverage were concentrated in the first and fourth quadrants. These findings suggested the differences between the two samples contributed to subtle flavor variations observed between the early and late stages of storage. Similarly, Jo et al. [65] utilized PCA graphs to unveil the disparities in active aroma components within skimmed dairy products subjected to diverse ultra-pasteurization processes. The tendency of convergence towards the centroid is also observed. The flavor profile of the lactic acid beverages during storage is depicted in Figure 7D. Variations in peak intensity at the same retention time reflect differences in the concentrations of various substances, whereas the number and area of peaks indicate quantitative differences in flavor compounds.

#### 3.7.2. Variations in Volatile Flavor Substance Contents in Beverages During Storage

The variations in volatile flavor substances between the NJAU-01 lactic acid beverage and Y during storage were investigated qualitatively using SPME-GC-MS (Table 3). On day 0, the total concentration of flavor substances in NJAU-01 and Y were 551.10 μg/L and 960.12 μg/L, respectively. By day 21 of storage, these values decreased to 356.65 μg/L and 757.11 μg/L.

The fatty acid content of both beverages increased during storage. Fatty acids originated from the degradation of lactose and amino acids, especially short-chain fatty acids [66]. Short-chain and medium-chain fatty acids, due to their low perception thresholds, significantly impact the aroma of dairy products, particularly butyric acid, caproic acid, and caprylic acid [67]. In the NJAU-01 beverage, n-hexanoic acid plays a major role in enhancing acidity, while caprylic acid imparts a stronger acidity in the Y beverage.

The two beverages exhibited significant disparity in the quantity of the alcohols. Alcohols are another important flavor component in dairy products, typically generated through the reduction in aldehydes and amino acid metabolism. The presence of volatile alcohols, particularly unsaturated ones or those present in higher concentrations, can significantly impact the flavor profile of dairy products due to their high odor threshold. In the NJAU-01 beverage, 1-nonanol and n-hexanol contributed distinct rose and fruit flavors. In the Y beverage, 1-nonanol, nerol, and linalool contributed rose and orange flavors.

Esters produced by hydrolysis and the esterification of hydroxy fatty acid triglycerides [68] contributed to the typical flavors of cow’s milk and cream. In the NJAU-01 beverage, δ-decalactone was detected and contributed a coconut and sweet flavor to the overall aroma [40], while in the Y beverage, ethyl butyrate contributed a significant pineapple flavor.

Alkanes such as heptane, octane, nonane, decane, and undecane are generally present in yogurt. However, the concentrations of alkanes with high thresholds are almost irrelevant to the flavor [69]. The alkane content in the NJAU-01 beverage was relatively low and had a minor flavor contribution, whereas limonene and terpinene, as food flavorings, significantly enhanced the aroma and taste of the beverage.

The results of Liu et al. [70] showed that the mild fermentation of Lactobacillus plantarum LP 56 increased the contents of alcohols, acids, ketones, and esters, enhancing the aroma quality of walnut milk.

#### 3.7.3. Variations in Odor Activity Values (OAVs) of Beverages During Storage

To evaluate the contribution of volatile compounds to the overall aroma profile of the lactic acid beverages, the odor activity value (OAV) was further calculated (Table 4). An OAV > 1 indicated an impact from volatile fragrances on the aroma of lactic acid beverages. Specifically, the NJAU-01 beverage contained 7 substances and Y contained 6 substances with OAVs that exceeded 1. The OAV analysis indicated that alcohols, aldehydes, esters, acids, and terpenes were the primary flavor-contributing compounds in beverages. Liu et al. [71] analyzed the OAVs and concluded that 2, 3-butanedione, butyric acid, caproic acid, and δ-decalactone were the key aroma-active substances in fermented milk. Aldehydes and ketones make significant contributions to the flavor characteristics of heat-treated dairy products [72]. The main forms of ketones were methyl ketones, which were derivatives of free fatty acids. The ketones exhibit distinctive aromas, such as fruity and floral notes, which can enhance the flavor profile of dairy products. Moreover, they possess a low perception threshold, as demonstrated by the typical volatile compound 2-heptanone A small quantity of 2-heptanone and 2-nonanone were detected in the NJAU-01 lactic acid beverage, while carvone and methyl heptenone were detected in the Y beverage. Studies have indicated that 2-heptanone and 2-nonanone contribute to the sensory attributes of dairy and oily aromas [73]. The content of ketone substances measured was relatively low since skimmed milk powder contained fewer lipids. Aldehyde compounds with very low perception thresholds make a prominent contribution to the flavor of fermented milk. Low concentrations of aldehydes with herbal aromas are conducive to the fresh flavor of milk [74]. The presence of nonanal was detected in the NJAU-01 lactic acid beverage, while isovaleraldehyde was found in the Y beverage, with both contributing honey and floral flavors to the lactic acid beverages.

## 4. Conclusions

NJAU-01 demonstrated the ability to effectively alleviate MDA-induced oxidative stress, with a maximum tolerance of 40 mM MDA. Under this stress condition, NJAU-01 exhibited enhanced in vitro antioxidant capacity and an improved antioxidant enzyme system. The optimized process resulted in a live bacterial lactic acid beverage that received favorable sensory evaluations, exhibited a higher total antioxidant capacity, and effectively eliminated MDA, compared to the Y beverage. The storage stability of the NJAU-01 beverage at 4 °C was determined to be 21 days. During this period, the acidity and viscosity of the beverage remained stable, while it also maintained favorable sensory attributes and exhibited a robust resistance to lipid peroxidation. Notable contributors to the flavor profile of the NJAU-01 lactic acid beverage included 2,3-butanedione, 2,3-entanedione, 2,3-hexanedione, 2,3-heptanedione, 2,3-octanedione, and 2,3-nonanedione. Our findings were helpful for establishing a theoretical foundation for industrial production and were beneficial for ensuring the quality of the NJAU-01 lactic acid beverage during storage.

The NJAU-0l strain demonstrated restricted acid production capability, which, in conjunction with other strains exhibiting robust acid production, could be harnessed for its high resistance to peroxidation in beverages. Future research could delve deeper into the role of the NJAU-01 beverage in mitigating lipid oxidation by conducting experiments using a mouse model. In response to the increasing market demand, probiotic products are evolving beyond conventional formats to include innovative core offerings such as capsules, membrane-coated soft capsules, and liquid probiotics. The strain NJAU-01, known for its specific health benefits, can be integrated into these novel non-dairy probiotic products to meet consumer demands for convenience and diversity.

## Figures and Tables

**Table 1:** Intensity of 270 to 130 kDA protein bands.

Sample	+Mercaptoethanol	−Mercaptoethanol
NJAU-01	295,378	468,606
0 h	386,947	507,988
4 h	423,444	618,450
12 h	417,184	565,550
24 h	411,552	462,140
26 h	394,399	466,192
32 h	386,211	482,419
44 h	339,259	481,844

**Table 2:** (**A**) Response surface design scheme and results. (**B**) Analysis of variance of quadratic model of sensory evaluation. (**C**) Analysis of variance of quadratic model of total antioxidant capacity.

(**A**)						
**Number**	**(A) Inoculum**	**(B) Fermentation Time**	**(C) Sugar Addition**		**Sensory Score**	**T-AOC** **(mM/g prot)**
1	1	0	−1		71.56	7.40
2	−1	1	0		61.10	6.57
3	1	1	0		78.34	7.16
4	0	0	0		84.42	7.07
5	0	0	0		82.48	7.16
6	0	0	0		83.04	7.18
7	0	−1	−1		68.06	6.63
8	0	0	0		83.16	7.20
9	1	−1	0		85.28	6.96
10	−1	0	−1		59.14	6.66
11	0	0	0		85.52	7.16
12	0	1	1		78.26	6.79
13	0	1	−1		60.86	6.78
14	−1	0	1		64.50	6.75
15	1	0	1		86.92	7.50
16	0	−1	1		72.70	6.46
17	−1	−1	0		59.64	6.48
(**B**)						
**Source**	**Sum of Squares**	**df**	**Mean Squares**	**F-Value**	***p*-Value**	**Significance**
Model	1690.20	9	187.80	81.22	<0.0001	***
A-Inoculum	755.05	1	755.05	326.56	<0.0001	***
B-Fermentation time	6.34	1	6.34	2.74	0.1418	
C-sugar addition	228.55	1	228.55	98.85	<0.0001	***
AB	17.64	1	17.64	7.63	0.0280	*
AC	25.00	1	25.00	10.81	0.0133	*
BC	40.70	1	40.70	17.60	0.0041	**
A^2^	153.45	1	153.45	66.37	<0.0001	***
B^2^	183.24	1	183.24	79.25	<0.0001	***
C^2^	215.67	1	215.67	93.28	<0.0001	***
Residual	16.18	7	2.31			
Lack of Fit	10.14	3	3.38	2.24	0.2262	
Pure Error	6.04	4	1.51			
Cor Total	1706.38	16				
Model	R^2^ = 0.9905			R^2^Adj = 0.9783		
(**C**)						
**Source**	**Sum of Squares**	**df**	**Mean Squares**	**F-Value**	***p*-Value**	**Significance**
Model	1.60	9	0.18	23.52	0.0002	***
A-Inoculum	0.82	1	0.82	108.12	<0.0001	***
B-Fermentation time	0.076	1	0.076	10.11	0.0155	*
C-Sugar addition	0.0003	1	0.0003	0.040	0.8475	
AB	0.0035	1	0.0035	0.46	0.5187	
AC	4.000 × 10^−6^	1	4.000 × 10^−6^	0.0005	0.9823	
BC	0.0078	1	0.0078	1.04	0.3421	
A^2^	0.0029	1	0.0029	0.39	0.5529	
B^2^	0.63	1	0.63	83.57	<0.0001	***
C^2^	0.043	1	0.043	5.74	0.0478	
Residual	0.053	7	0.0075			
Lack of Fit	0.042	3	0.014	5.32	0.0702	
Pure Error	0.011	4	0.0026			
Cor Total	1.65	16				
Model	R^2^ = 0.9680			R^2^Adj = 0.9268		

Note: T-AOC (total antioxidant capacity); *** indicates a highly significant difference (*p* < 0.001); ** indicates a very significant difference (*p* < 0.01); * indicates a significant difference (*p* < 0.05).

**Table 3:** Changes in volatile flavor substances in lactic acid bacteria beverages during storage.

		A0 μg/L	Y0 μg/L	AM μg/L	YM μg/L
Ketones	2-heptanone	60.40 ± 4.93	/	27.09 ± 1.70	/
	2-tridecanone	15.39 ± 2.04	/	4.47 ± 0.16	/
	2-undecanone	23.81 ± 2.24	/	16.02 ± 2.99	/
	Isophorone	2.83 ± 0.45	/	3.13 ± 0.51	/
	2-nononone	43.84 ± 0.51	/	14.86 ± 2.58	/
Aldehydes	2, 5-dimethylbenzaldehyde	1.22 ± 0.14	/	1.50 ± 0.34	1.02 ± 0.45
	Acetaldehyde-2, 3-butane diol acetal	0.66 ± 0.12	/	/	/
	3-hydroxybutyraldehyde	13.54 ± 2.62	/	12.58 ± 0.55	/
	Heptanal	5.71 ± 1.39	/	4.33 ± 0.20	/
	Isovaleraldehyde	4.29 ± 0.16	/	8.18 ± 1.24	/
	Nonanal	1.25 ± 0.17	/	/	2.84 ± 0.28
	Butanedial	/	/	2.78 ± 0.57	/
	2-methylvaleraldehyde	0.60 ± 0.05	/	/	/
	Citral	/	6.95 ± 1.41	/	/
	Perillaldehyde	/	6.49 ± 0.72	/	2.74 ± 0.10
	(Z)-3, 7-dimethyl-2, 6-octanedienal	/	5.63 ± 1.19	/	/
	Octyl propanediol acetal	/	16.06 ± 1.64	/	/
Acids	Acetic acid	41.13 ± 11.02	/	66.24 ± 10.08	/
	Malonic acid	13.30 ± 2.43	/	8.54 ± 2.15	/
	L-lactic acid	18.98 ± 2.92	/	1.69 ± 0.04	/
	2-ethylbutyric acid	2.42 ± 0.14	/	/	/
	N-hexylic acid	6.15 ± 0.29	/	/	/
	Octanoic acid	/	8.29 ± 1.59	/	/
	Caproic acid	/	4.19 ± 1.64	/	4.88 ± 0.10
	N-decanoic acid	/	2.37 ± 1.08	/	/
Alcohols	Linalool	/	115.59 ± 11.20	/	97.59 ± 15.68
	decyl alcohol	/	79.33 ± 13.71	/	67.63 ± 0.15
	4-terpenol	/	25.82 ± 8.32	/	12.30 ± 2.34
	Geraniol	/	21.39 ± 3.40	/	9.64 ± 0.38
	1-octanol	/	9.35 ± 3.24	/	3.54 ± 0.69
	2-nonyl alcohol	37.60 ± 4.58	0.84 ± 0.16	37.00 ± 1.98	/
	2-hydrazyl ethanol	126.42 ± 12.48	/	41.12 ± 1.55	/
	2-tetradecyl alcohol	14.21 ± 2.71	/	/	/
	2-heptanol	24.32 ± 4.91	/	20.37 ± 2.57	/
	Furfuryl alcohol	12.05 ± 1.31	4.40 ± 0.61	12.13 ± 1.72	3.96 ± 0.46
	1-nonyl alcohol	7.30 ± 0.28	14.22 ± 3.01	4.73 ± 1.23	/
	4-methyl-2-hexanol	8.26 ± 2.30	/	7.24 ± 0.66	/
	Butane-1,2, 3-triol	2.22 ± 0.39	/	/	/
	Isoamylol	3.33 ± 1.40	/	/	/
	Hexyl alcohol	2.88 ± 0.70	/	1.43 ± 0.22	/
	Propylene glycol	12.98 ± 0.78	/	11.30 ± 2.30	/
	2-ethyl hexanol	15.74 ± 2.38	/	/	/
	5-ethyl-2-nonanol	7.01 ± 1.33	/	4.75 ± 0.12	/
	1,2, 3-butanediol	0.90 ± 0.04	/	/	/
	2-pentadecyl alcohol	/	/	6.53 ± 0.29	/
	4-pentene-2-ol	/	/	6.84 ± 2.07	/
	2-decanol	/	/	3.38 ± 0.27	/
	2, 5-dimethyl-3-hexanol	/	/	2.73 ± 0.11	/
	2-ethylxyethanol	/	/	0.72 ± 0.15	/
	4-butoxy-1-butanol	/	/	0.98 ± 0.27	/
	Eudesmol	/	2.71 ± 0.98	/	137.17 ± 7.29
	Fenchol	/	5.07 ± 1.15	/	2.11 ± 0.32
	Thujyl alcohol	/	2.45 ± 0.87	/	/
	Nerol	/	2.37 ± 0.45	/	0.89 ± 0.13
	L-undecanol	/	2.84 ± 0.67	/	7.99 ± 0.30
	p-menthyl-3-ene-1-ol	/	1.60 ± 0.65	/	0.51 ± 0.08
	Laurinol	/	2.12 ± 0.53	/	/
	3, 7-dimethyl-3, 6-octadiene-1-ol	/	4.38 ± 1.28	/	5.33 ± 1.70
	2, 3-dimethylcyclohexanol	/	1.02 ± 0.08	/	0.49 ± 0.19
	Perilla alcohol	/	0.75 ± 0.30	/	/
	Acetone alcohol	/	0.72 ± 0.09	/	/
Esters	Ethyl caprate	2.76 ± 0.41	/	3.46 ± 0.78	/
	Butyl decyllactone	0.73 ± 0.15	/	/	/
	Octyl ethylene glycol mono-n-dodecyl ester	0.42 ± 0.13	/	/	/
	Pentyl acrylate	/	/	0.77 ± 0.09	/
	Ethyl orthoformate	0.74 ± 0.26	/	0.75 ± 0.07	/
	2-Methoxyethyl acetate	1.15 ± 0.63	/	/	/
	Ethyl caprylate	0.55 ± 0.08	/	/	/
	Ethyl caprylate	/	24.73 ± 4.41	/	12.01 ± 1.72
	Geranyl acetate	/	21.80 ± 4.25	/	10.75 ± 0.82
	Isoamyl acetate	/	6.52 ± 2.53	/	2.88 ± 0.12
	Octyl acetate	/	4.28 ± 1.11	/	/
	Allyl hexanoate	/	3.51 ± 0.51	/	1.55 ± 0.28
	Linalyl acetate	/	1.96 ± 0.33	/	0.80 ± 0.03
Alkyl alkenes	L-methoxyoctane	1.23 ± 0.16	/	2.81 ± 0.71	/
	2,3, 3-trimethylpentane	3.28 ± 0.22	/	5.79 ± 0.87	/
	N-Hexadecane	5.33 ± 0.35	/	3.13 ± 0.53	/
	2, 3-epoxy-2-methylhexane	1.19 ± 0.29	/	3.76 ± 1.52	/
	Tridecane	/	/	0.78 ± 0.03	/
	Limonene	/	426.63 ± 35.03	/	328.72 ± 18.45
	Terpinene	/	59.04 ± 13.08	/	9.10 ± 1.56
	Beta-pinene	/	35.88 ± 4.99	/	14.74 ± 0.76
	Myrcene	/	15.69 ± 2.77	/	5.83 ± 0.47
	Terpinolene	/	10.50 ± 2.83	/	6.41 ± 1.25
	Terpinolene	/	2.57 ± 0.74	/	3.67 ± 0.40
Else	2-methylpiperazine	2.97 ± 0.42	/	2.69 ± 0.15	/
Total		551.10 ± 7.49	960.12 ± 68.48	356.65 ± 20.61	757.11 ± 91.96

The symbol “/” indicates that the volatile flavor substances were either undetectable or incomputable. A0 denotes the NJAU-01 beverage stored for 0 days, AM represents the NJAU-01 beverage stored for 21 days, Y0 signifies Y stored for 0 days, and YM stands for Y stored for 21 days.

**Table 4:** The aroma activity value of the flavor compounds of the milk drinks during the storage period.

Substance	Flavor Characteristics	Threshold Value (mg/L)	A0	Y0	AM	YM
Heptan-2-one	Cream, fruit flavor	140	0.43	/	0.19	/
2-undecone	Orange, grass flavor	80	0.29	/	0.26	/
2-nononone	Grass, fruit flavor	100	0.44	/	0.15	/
Heptanal	Fresh fruity scent	3000	0.02	/	0.01	/
Isovaleral	Floral and fruity	3.6	1.19	/	2.27	/
Nonanal	Floral, citrus, honey aromas	1	1.25	/	/	2.85
Acetic acid	Acetic	99	0.41	/	0.67	/
N-hexylic acid	Acetic	0.98	6.27	/	/	/
Octanoic acid	Acetic	3	/	2.76	/	/
N-capric acid	/	10	/	0.24	/	/
Linalool	Floral and fruity	6	/	19.27	/	16.27
Geraniol	Rose flower fragrance	26	/	0.82	/	0.37
2-nonyl alcohol	Rose and orange Fragrance	25	1.5	0.034	1.48	/
2-heptanol	Soil, oil	55	0.4	/	0.4	/
Furfuralcohol	Coffee, nutty	5000	0.01	0.01	0.02	0.01
1-nonyl alcohol	Rose and orange fragrance	0.045	162.29	315.83	105.04	/
Hexyl alcohol	Fruity	0.0056	514.44	/	255.27	/
Nerol	Rose flower fragrance	0.01	/	237.16	/	88.82
Butyl decyllactone	Peach flavor	0.06	12.13	/	/	/
Ethyl n-caproate	Tropical fruit fragrance	1	0.74	/	0.75	/
Linalool	Floral and fruity	6	/	19.27	/	16.27
Isoamyl acetate	Banana, pear flavor	73	/	0.09	/	0.05
Limonene	Lemon, mint flavor	200	/	2.13	/	1.64
Terpinene	Herbaceous	108	/	0.55	/	0.09
β-Pinene	/	140	/	0.26	/	0.11
Myrcene	Light balsam flavor	100	/	0.16	/	0.06
α-pinene	/	6	/	0.1	/	/

The symbol “/” indicates that the OAV was either undetectable or incomputable. A0 denotes the NJAU-01 beverage stored for 0 days, AM represents the NJAU-01 beverage stored for 21 days, Y0 signifies Y stored for 0 days, and YM stands for Y stored for 21 days.

## Data Availability

The original contributions presented in this study are included in Supplementary Material. Further inquiries can be directed to the corresponding author.

## Supplementary Materials

The following supporting information can be downloaded at: https://www.mdpi.com/article/10.3390/foods14010052/s1. Figure S1: Growth curves of NJAU-01 under various MDA concentrations; Table S1: Sensory evaluation standard for lactic acid beverages.

## References

[1] Anwer M., Wei M.Q. (2024). Harnessing the power of probiotic strains in functional foods: Nutritive, therapeutic, and next-generation challenges. Food Sci. Biotechnol..

[2] Zommiti M., Feuilloley M.G.J., Connil N. (2020). Update of Probiotics in Human World: A Nonstop Source of Benefactions till the End of Time. Microorganisms.

[3] Wang Y.Y., Wang B.K., Zhan X.A., Wang Y.B., Li W.F. (2022). Effects of glucose oxidase and its combination with B. amyloliquefaciens SC06 on intestinal microbiota, immune response and antioxidative capacity in broilers. Animal.

[4] Amanatidou A., Smid E.J., Bennik M.H.J., Gorris L.G.M. (2001). Antioxidative properties of upon exposure to elevated oxygen concentrations. FEMS Microbiol. Lett..

[5] Sun J., Hu X.L., Le G.W., Shi Y.H. (2010). Lactobacilli prevent hydroxy radical production and inhibit and growth in system mimicking colon fermentation. Lett. Appl. Microbiol..

[6] Kim K.J., Kyung S., Jin H., Im M., Kim J.W., Kim H.S., Jang S.E. (2023). Lactic Acid Bacteria Isolated from Human Breast Milk Improve Colitis Induced by 2,4,6-Trinitrobenzene Sulfonic Acid by Inhibiting NF-κB Signaling in Mice. J. Microbiol. Biotechnol..

[7] Nazir M., Arif S., Khan R.S., Nazir W., Khalid N., Maqsood S. (2019). Opportunities and challenges for functional and medicinal beverages: Current and future trends. Trends Food Sci. Technol..

[8] Shudong P., Guo C.Q., Wu S.J., Cui H.C., Suo H.Y., Duan Z. (2022). Bioactivity and metabolomics changes of plant-based drink fermented by Bacillus coagulans VHProbi C08. LWT-Food Sci. Technol..

[9] Kadyan S., Rashmi H.M., Pradhan D., Kumari A., Chaudhari A., Deshwal G.K. (2021). Effect of lactic acid bacteria and yeast fermentation on antimicrobial, antioxidative and metabolomic profile of naturally carbonated probiotic whey drink. LWT-Food Sci. Technol..

[10] Zhang Y.Z., Liu P.P., Fu H., Wang D.D., Zhao D., Zhang J.C., Wang C.T., Li M. (2022). Effects of fermentation supernatant on skin aging caused by oxidative stress. J. Funct. Foods.

[11] Medeiros M.H.G. (2019). DNA Damage by Endogenous and Exogenous Aldehydes. J. Braz. Chem. Soc..

[12] Tavakoli M., Najafi M.B.H., Mohebbi M. (2019). Effect of the milk fat content and starter culture selection on proteolysis and antioxidant activity of probiotic yogurt. Heliyon.

[13] Ge Q.F., Ge P.W., Jiang D.L., Du N., Chen J.H., Yuan L.M., Yu H., Xu X., Wu M.G., Zhang W.G. (2018). A novel and simple cell-based electrochemical biosensor for evaluating the antioxidant capacity of strains isolated from Chinese dry-cured ham. Biosens. Bioelectron..

[14] Ge Q.F., Yang B., Liu R., Jiang D.L., Yu H., Wu M.G., Zhang W.G. (2021). Antioxidant activity of Lactobacillus plantarum NJAU-01 in an animal model of aging. BMC Microbiol..

[15] Chen L., Liu R., Li S.Y., Yu H., Ge Q.F. (2023). Metabolism of hydrogen peroxide by Lactobacillus plantarum NJAU-01: A proteomics study. Food Microbiol..

[16] Jin D.X., Jia C.Y., Yang B., Wu Y.H., Chen L., Liu R., Wu M.G., Yu H., Ge Q.F. (2024). The ameliorative mechanism of NJAU-01 against D-galactose induced oxidative stress: A hepatic proteomics and gut microbiota analysis. Food Funct..

[17] Ge Q.F., Chen S., Liu R., Chen L., Yang B., Yu H., Wu M.G., Zhang W.G., Zhou G.H. (2019). Effects of NJAU-01 on the protein oxidation of fermented sausage. Food Chem..

[18] Kullisaar T., Zilmer M., Mikelsaar M., Vihalemm T., Annuk H., Kairane C., Kilk A. (2002). Two antioxidative lactobacilli strains as promising probiotics. Int. J. Food Microbiol..

[19] Kusuhara S., Ito M., Sato T., Yokoi W., Yamamoto Y., Harada K., Ikemura H., Miyazaki K. (2018). Intracellular GSH of Streptococcus thermophilus shows anti-oxidative activity against low-density lipoprotein oxidation in vitro and in a hyperlipidaemic hamster model. Benef. Microbes.

[20] Cataldo P.G., Villegas J.M., de Giori G.S., Saavedra L., Hebert E.M. (2020). Enhancement of gamma-aminobutyric acid (GABA) production by Lactobacillus brevis CRL 2013 based on carbohydrate fermentation. Int. J. Food Microbiol..

[21] Li W.Y., Gao L.E., Huang W.K., Ma Y., Muhammad I., Hanif A., Ding Z.T., Guo X.S. (2022). Antioxidant properties of lactic acid bacteria isolated from traditional fermented yak milk and their probiotic effects on the oxidative senescence of Caenorhabditis elegans. Food Funct..

[22] Saleena L.A.K., Chandran D., Geetha R., Radha R., Sathian C.T., Kumar M., Sureshkumar R., Marthandan V., Radha (2023). Optimization and Identification of Lactic Acid Bacteria with Higher Mannitol Production Potential. Indian J. Anim. Res..

[23] Wu W., Zhang C.M., Hua Y.F. (2009). Structural modification of soy protein by the lipid peroxidation product malondialdehyde. J. Sci. Food Agric..

[24] Tang W., Xing Z.Q., Li C., Wang J.J., Wang Y.P. (2017). Molecular mechanisms and antioxidant effects of MA2. Food Chem..

[25] Liu R., Li S.Y., Yang B., Chen L., Ge Q.F., Xiong G.Y., Yu H., Wu M.A., Zhang W.A. (2021). Investigation of the antioxidant capacity of cell-free extracts from NJAU-01 obtained by different cell disruption methods. LWT-Food Sci. Technol..

[26] Li S.Y., Zhao Y.J., Zhang L., Zhang X., Huang L., Li D., Niu C.H., Yang Z.N., Wang Q. (2012). Antioxidant activity of strains isolated from traditional Chinese fermented foods. Food Chem..

[27] Padilla P., Andrade M.J., Peña F.J., Rodríguez A., Estévez M. (2022). Molecular mechanisms of the disturbance caused by malondialdehyde on probiotic PL503. Microb. Biotechnol..

[28] Lee M., Song J.H., Choi E.J., Yun Y.R., Lee K.W., Chang J.Y. (2021). UPLC-QTOF-MS/MS and GC-MS Characterization of Phytochemicals in Vegetable Juice Fermented Using Lactic Acid Bacteria from Kimchi and Their Antioxidant Potential. Antioxidants.

[29] Zheng L., Zhao M.M., Xiao C.Q., Zhao Q.Z., Su G.W. (2016). Practical problems when using ABTS assay to assess the radical-scavenging activity of peptides: Importance of controlling reaction pH and time. Food Chem..

[30] Shen Y.B., Zhang H., Cheng L.L., Wang L., Qian H.F., Qi X.G. (2016). In vitro and in vivo antioxidant activity of polyphenols extracted from black highland barley. Food Chem..

[31] Quatravaux S., Remize F., Bryckaert E., Colavizza D., Guzzo J. (2006). Examination of lactate metabolism side effects in relation to the modulation of aeration parameters. J. Appl. Microbiol..

[32] Zhu L.J., Xiong H.G., Huang X., Guyonnet V., Ma M.H., Chen X.Q., Zheng Y.T., Wang L.M., Hu G. (2022). Identification and molecular mechanisms of novel antioxidant peptides from two sources of eggshell membrane hydrolysates showing cytoprotection against oxidative stress: A combined in silico and in vitro study. Food Res. Int..

[33] Ghani M.A., Barril C., Bedgood D.R., Prenzler P.D. (2017). Measurement of antioxidant activity with the thiobarbituric acid reactive substances assay. Food Chem..

[34] Lee B.J., Kim J.S., Kang Y.M., Lim J.H., Kim Y.M., Lee M.S., Jeong M.H., Ahn C.B., Je J.Y. (2010). Antioxidant activity and γ-aminobutyric acid (GABA) content in sea tangle fermented by BJ20 isolated from traditional fermented foods. Food Chem..

[35] Zhao L.L., Zhang M., Wang H.X., Devahastin S. (2020). Effect of carbon dots in combination with aqueous chitosan solution on shelf life and stability of soy milk. Int. J. Food Microbiol..

[36] Lee S.M., Hwang Y.R., Kim M.S., Chung M.S., Kim Y.S. (2019). Comparison of Volatile and Nonvolatile Compounds in Rice Fermented by Different Lactic Acid Bacteria. Molecules.

[37] Zhang L., Mi S., Liu R.B., Sang Y.X., Wang X.H. (2020). Evaluation of Volatile Compounds during the Fermentation Process of Yogurts by Streptococcus thermophilus Based on Odor Activity Value and Heat Map Analysis. Int. J. Anal. Chem..

[38] Zhao M., Ma H.R., Hou Y.Q., Li J., Zou T.T., Zhang D.J., Wen R., Li H.L., Song H.L. (2022). Characterization of Key Odor-Active Off-Flavor Compounds in Aged Pasteurized Yogurt by Sensory-Directed Flavor Analysis. J. Agric. Food Chem..

[39] Dan T., Hu H.M., Li T., Dai A.N., He B.B., Wang Y.A. (2022). Screening of mixed-species starter cultures for increasing flavour during fermentation of milk. Int. Dairy J..

[40] Liu A., Zhang H.W., Liu T.J., Gong P.M., Wang Y.W., Wang H.Z., Tian X.Y., Liu Q.Q., Cui Q.Y., Xie X. (2022). Aroma classification and flavor characterization of fermented milk by HS-GC-IMS and HS-SPME-GC-TOF/MS. Food Biosci..

[41] Fritz K.S., Petersen D.R. (2013). An overview of the chemistry and biology of reactive aldehydes. Free. Radic. Biol. Med..

[42] Su L.J., Zhang J.H., Gomez H., Murugan R., Hong X., Xu D.X., Jiang F., Peng Z.Y. (2019). Reactive Oxygen Species-Induced Lipid Peroxidation in Apoptosis, Autophagy, and Ferroptosis. Oxidative Med. Cell. Longev..

[43] Basu Thakur P., Long A.R., Nelson B.J., Kumar R., Rosenberg A.F., Gray M.J. (2019). Complex Responses to Hydrogen Peroxide and Hypochlorous Acid by the Probiotic Bacterium Lactobacillus reuteri. mSystems.

[44] Vandemoortele A., Heynderickx P.M., Leloup L., De Meulenaer B. (2021). Kinetic modeling of malondialdehyde reactivity in oil to simulate actual malondialdehyde formation upon lipid oxidation. Food Res. Int..

[45] Vandemoortele A., Babat P., Yakubu M., De Meulenaer B. (2017). Reactivity of Free Malondialdehyde during In Vitro Simulated Gastrointestinal Digestion. J. Agric. Food Chem..

[46] Lv Y., Chen L., Wu H., Xu X., Zhou G., Zhu B., Feng X. (2019). (-)-Epigallocatechin-3-gallate-mediated formation of myofibrillar protein emulsion gels under malondialdehyde-induced oxidative stress. Food Chem..

[47] Zhang Z., Xiong Z., Lu S., Walayat N., Hu C., Xiong H. (2020). Effects of oxidative modification on the functional, conformational and gelling properties of myofibrillar proteins from Culter alburnus. Int. J. Biol. Macromol..

[48] Wu X.J., Li F., Wu W. (2020). Effects of rice bran rancidity on the oxidation and structural characteristics of rice bran protein. LWT-Food Sci. Technol..

[49] Gatellier P., Santé-Lhoutellier V., Portanguen S., Kondjoyan A. (2009). Use of meat fluorescence emission as a marker of oxidation promoted by cooking. Meat Sci..

[50] Sobral M.M.C., Casal S., Faria M.A., Cunha S.C., Ferreira I.M.P.L.V.O. (2020). Influence of culinary practices on protein and lipid oxidation of chicken meat burgers during cooking and gastrointestinal digestion. Food Chem. Toxicol..

[51] Ulrich K., Jakob U. (2019). The role of thiols in antioxidant systems. Free Radic. Biol. Med..

[52] Arcanjo N.O., Andrade M.J., Padilla P., Rodríguez A., Madruga M.S., Estévez M. (2019). Resveratrol protects against HO- induced oxidative stress and stimulates antioxidant defenses through upregulation of the gene. Free Radic. Biol. Med..

[53] Zhang H., Wang Z., Li Z.W., Wang K.D., Kong B.H., Chen Q. (2022). L-glycine and L-glutamic acid protect R1 against oxidative damage induced by hydrogen peroxide. Food Microbiol..

[54] Zhang L., Liao C.M., Yang Y.W., Wang Y.Z., Ding K., Huo D.Q., Hou C.J. (2019). Response of lipid biosynthesis into intracellular reactive oxygen species level under stress conditions. Bioresour. Technol..

[55] Kang R.F., Li R.N., Dai P.Y., Li Z.J., Li Y.S., Li C.M. (2019). Deoxynivalenol induced apoptosis and inflammation of IPEC-J2 cells by promoting ROS production. Environ. Pollut..

[56] Forman H.J., Zhang H.Q., Rinna A. (2009). Glutathione: Overview of its protective roles, measurement, and biosynthesis. Mol. Asp. Med..

[57] Jové M., Mota-Martorell N., Pradas I., Martín-Gari M., Ayala V., Pamplona R. (2020). The Advanced Lipoxidation End-Product Malondialdehyde-Lysine in Aging and Longevity. Antioxidants.

[58] Liu L., Qu X.W., Xia Q.N., Wang H.X., Chen P., Li X.D., Wang L.N., Yang W.S. (2018). Effect of on the antioxidant activity of Cheddar cheese during ripening and under simulated gastrointestinal digestion. LWT-Food Sci. Technol..

[59] Wang A.R., Hou K.R., Mu G.Q., Ma C.L., Tuo Y.F. (2021). Antioxidative effect of soybean milk fermented by Y16 on 2, 2 -azobis (2-methylpropionamidine) dihydrochloride (ABAP)-damaged HepG2 cells. Food Biosci..

[60] Shori A.B., Aljohani G.S., Al-zahrani A.J., Al-sulbi O.S., Baba A.S. (2022). Viability of probiotics and antioxidant activity of cashew milk-based yogurt fermented with selected strains of probiotic *Lactobacillus* spp. LWT-Food Sci. Technol..

[61] Fan X.K., Du L.H., Xu J., Shi Z.H., Zhang T., Jiang X.X., Zeng X.Q., Wu Z., Pan D.D. (2022). Effect of single probiotics CGMCC1.5956 and CGMCC1.5954 and their combination on the quality of yogurt as fermented milk. LWT-Food Sci. Technol..

[62] Zahrani A.J.A., Shori A.B. (2023). Viability of probiotics and antioxidant activity of soy and almond milk fermented with selected strains of probiotic. LWT-Food Sci. Technol..

[63] Shori A.B., Rashid F., Baba A.S. (2018). Effect of the addition of phytomix-3+mangosteen on antioxidant activity, viability of lactic acid bacteria, type 2 diabetes key-enzymes, and sensory evaluation of yogurt. LWT-Food Sci. Technol..

[64] Chi X.L., Shao Y.W., Pan M.H., Yang Q.Y., Yang Y., Zhang X.M., Ai N.S., Sun B.G. (2021). Distinction of volatile flavor profiles in various skim milk products via HS-SPME-GC-MS and E-nose (Mar, 10.1007/s00217-021-03730-0, 2021). Eur. Food Res. Technol..

[65] Jo Y., Benoist D.M., Barbano D.M., Drake M.A. (2018). Flavor and flavor chemistry differences among milks processed by high-temperature, short-time pasteurization or ultra-pasteurization. J. Dairy Sci..

[66] Bui A.T.H., Cozzolino D., Zisu B., Chandrapala J. (2020). Effects of high and low frequency ultrasound on the production of volatile compounds in milk and milk products—A review. J. Dairy Res..

[67] Toelstede S., Hofmann T. (2008). Sensomics mapping and identification of the key bitter metabolites in Gouda cheese. J. Agric. Food Chem..

[68] Wang J., Yang Z.J., Xu L.Y., Wang B., Zhang J.H., Li B.Z., Cao Y.P., Tan L. (2021). Key aroma compounds identified in Cheddar cheese with different ripening times by aroma extract dilution analysis, odor activity value, aroma recombination, and omission. J. Dairy Sci..

[69] Iranmanesh M., Ezzatpanah H., Akbari-Adergani B., Karimi Torshizi M.A. (2018). SPME/GC-MS characterization of volatile compounds of Iranian traditional dried Kashk. Int. J. Food Prop..

[70] Liu W., Pu X., Sun J., Shi X., Cheng W., Wang B. (2022). Effect of Lactobacillus plantarum on functional characteristics and flavor profile of fermented walnut milk. LWT.

[71] Liu A., Liu Q.Q., Bu Y.S., Hao H.N., Liu T.J., Gong P.M., Zhang L.W., Chen C., Tian H.X., Yi H.X. (2022). Aroma classification and characterization of subsp fermented milk. Food Chem. X.

[72] Pan M.H., Tong L.J., Chi X.L., Ai N.S., Cao Y.G., Sun B.G. (2019). Comparison of Sensory and Electronic Tongue Analysis Combined with HS-SPME-GC-MS in the Evaluation of Skim Milk Processed with Different Preheating Treatments. Molecules.

[73] Dursun A., Güler Z., Sekerli Y.E. (2017). Characterization of volatile compounds and organic acids in ultra-high-temperature milk packaged in tetra brik cartons. Int. J. Food Prop..

[74] De Santis D., Fidaleo M. (2022). Effect of aging pit on volatile compounds and sensory attributes of traditional Italian Fossa cheese. LWT-Food Sci. Technol..

