# Supplementary material for: Storage Stability Enhancement of Lactic Acid Beverage Using Anti-MDA Lactiplantibacillus plantarum NJAU-01: The Antioxidant’s Role
Source: Foods. 2024 Dec 27;14(1):52. doi: 10.3390/foods14010052 (PMC11720519; doi:10.3390/foods14010052)
Supplement: Supplementary file 1 [file foods-14-00052-s001.zip › foods-3351790-supplementary.pdf]

Figure S1

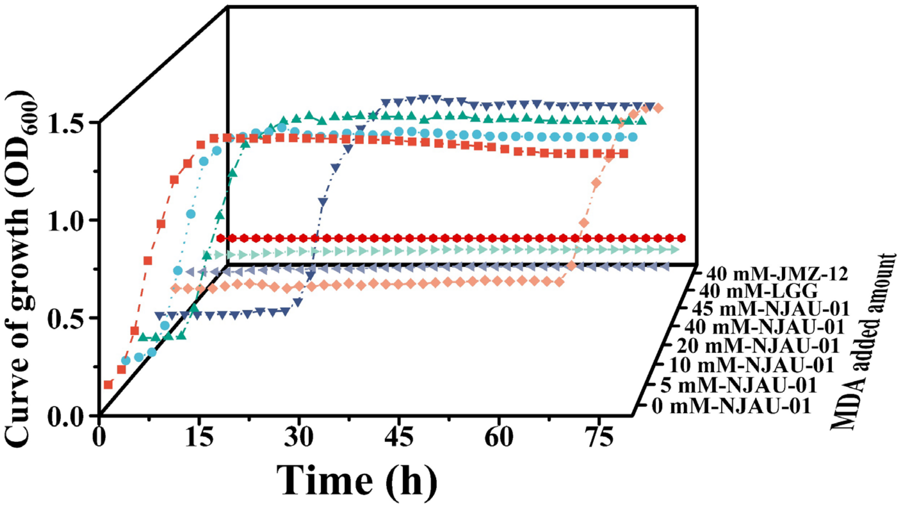

Figure S1. Growth curves of *L.* plantarum NJAU-01, *L. plantarum* JMZ-12 and *Lactobacillus rhamnosus* LGG under various MDA concentrations.

Table S1

Table S1. Sensory evaluation standard for lactic acid beverage.

Aspects	Standard	Assessment (score)
Color	uniform color, moderate brown color	Excellent（15~20）
	The brown color is slightly lighter or darker	Good（10~14）
	yellowish or slightly brown	Medium（5~9）
	Color milky white or earthy brow	Poor（0~4）
Aroma	Medium sweet and sour, smooth taste	Excellent（24~30）
	Sweet and sour is more moderate, smooth taste	Good（16~23）
	Slightly sour or sweet, slightly smooth taste	Medium（8~15）
	Too sour or too sweet, coarse and grainy	Poor（0~7）
Flavor	Intensely fermented flavor	Excellent（15~20）
	Less fermented flavor	Good（10~14）
	Light fermented flavor	Medium（5~9）
	No fermentation flavor	Poor（0~4）
Texture	Uniform and fine emulsion, no stratification phenomenon, no whey precipitation	Excellent（24~30）
	More uniform and fine emulsion, slight whey precipitation	Good（16~23）
	Uniform and fine emulsion, a small amount of whey precipitation	Medium（8~15）
	Thin emulsion, precipitated, lots of whey	Poor（0~7）
